# The endoplasmic reticulum-associated degradation machinery selectively degrades stress-induced TIN1 during stress recovery

**DOI:** 10.1093/plphys/kiaf489

**Published:** 2025-10-06

**Authors:** Yi Wang, Zhihui Ma, Jiarui Wu, Congcong Zhang, Yongwu Chen, Liangguang Leo Lin, Juan Mao, Jianjun Zhang, Linchuan Liu, Pengcheng Wang, Jianming Li

**Affiliations:** University of Chinese Academy of Science, Beijing 100004, China; Shanghai Center for Plant Stress Biology, The Center of Excellence for Molecular Plant Sciences, Chinese Academy of Sciences, Shanghai 201602, China; State Key Laboratory for Conservation and Utilization of Subtropical Agro-Bioresources, South China Agriculture University, Guangzhou 510642, China; Guangdong Key Laboratory for Innovative Development and Utilization of Forest Plant Germplasm, College of Forestry and Landscape Architecture, South China Agricultural University, Guangzhou, Guangdong 510642, China; State Key Laboratory for Conservation and Utilization of Subtropical Agro-Bioresources, South China Agriculture University, Guangzhou 510642, China; Guangdong Key Laboratory for Innovative Development and Utilization of Forest Plant Germplasm, College of Forestry and Landscape Architecture, South China Agricultural University, Guangzhou, Guangdong 510642, China; The Jockey Club STEM Laboratory of Plant Biology, Department of Biology, Hong Kong Baptist University, Kowloon, Hong Kong 999077, China; University of Chinese Academy of Science, Beijing 100004, China; Shanghai Center for Plant Stress Biology, The Center of Excellence for Molecular Plant Sciences, Chinese Academy of Sciences, Shanghai 201602, China; University of Chinese Academy of Science, Beijing 100004, China; Shanghai Center for Plant Stress Biology, The Center of Excellence for Molecular Plant Sciences, Chinese Academy of Sciences, Shanghai 201602, China; University of Chinese Academy of Science, Beijing 100004, China; Shanghai Center for Plant Stress Biology, The Center of Excellence for Molecular Plant Sciences, Chinese Academy of Sciences, Shanghai 201602, China; State Key Laboratory for Conservation and Utilization of Subtropical Agro-Bioresources, South China Agriculture University, Guangzhou 510642, China; Guangdong Key Laboratory for Innovative Development and Utilization of Forest Plant Germplasm, College of Forestry and Landscape Architecture, South China Agricultural University, Guangzhou, Guangdong 510642, China; State Key Laboratory for Conservation and Utilization of Subtropical Agro-Bioresources, South China Agriculture University, Guangzhou 510642, China; Guangdong Key Laboratory for Innovative Development and Utilization of Forest Plant Germplasm, College of Forestry and Landscape Architecture, South China Agricultural University, Guangzhou, Guangdong 510642, China; State Key Laboratory for Conservation and Utilization of Subtropical Agro-Bioresources, South China Agriculture University, Guangzhou 510642, China; Guangdong Key Laboratory for Innovative Development and Utilization of Forest Plant Germplasm, College of Forestry and Landscape Architecture, South China Agricultural University, Guangzhou, Guangdong 510642, China; Institute of Advanced Biotechnology, Institute of Homeostatic Medicine, School of Medicine, Southern University of Science and Technology, Shenzhen 518055, China; The Jockey Club STEM Laboratory of Plant Biology, Department of Biology, Hong Kong Baptist University, Kowloon, Hong Kong 999077, China; Department of Molecular, Cellular, and Developmental Biology, University of Michigan, Ann Arbor, MI 48109, USA; AoE Centre for Plant Vacuole Biology and Biotechnology, The Chinese University of Hong Kong, Shatin, Hong Kong 999077, China

## Abstract

The unfolded protein response (UPR) signaling pathway is activated by the accumulation of misfolded proteins in the endoplasmic reticulum (ER) and stimulates the production of ER chaperones to restore ER proteostasis. However, how UPR-induced proteins return to their pre-stress levels upon removal of ER stress remains unknown. TUNICAMYCIN-INDUCED 1 (TIN1) is an Arabidopsis (*Arabidopsis thaliana*) protein that is normally expressed in pollen but is rapidly induced by ER stress in vegetative tissues. Here, we show that the ER-stress-induced TIN1 is rapidly degraded in the UPR recovery phase. We found that TIN1 degradation depends on its asparagine-linked glycans and requires both EMS-mutagenized bri1 suppressor 5 (EBS5) and EBS6 for its recruitment to the ER-associated degradation (ERAD) complex. Loss-of-function mutations in the core component of this Arabidopsis ERAD complex greatly stabilize TIN1. Interestingly, 2 other UPR-induced proteins that are coexpressed with TIN1 remained stable upon ER-stress removal, suggesting that rapid degradation during the stress-recovery phase likely applies to a subset of UPR-induced proteins. Further investigation is needed to uncover the mechanisms by which the ERAD machinery selectively degrades UPR-induced ER proteins.

## Teaser

Plants use a selective degradation process to eliminate stress-induced proteins from the endoplasmic reticulum, supporting recovery and maintaining protein homeostasis after stress conditions.

## Introduction

The endoplasmic reticulum (ER) is an essential eukaryotic cellular organelle that plays an important role in calcium homeostasis, lipid biosynthesis, and the folding, modification, complex assembly, and transport of a wide range of transmembrane and secretory proteins (Ferro-Novick et al. 2013). Because protein folding is an error-prone process that can be easily disturbed by various cellular stresses, misfolded proteins often accumulate in the ER lumen, causing the so-called ER folding stress that interferes with the normal secretory processes crucial for cell survival. As a result, a stress response pathway, widely known as the UPR for unfolded protein response, is activated to promote the production of protein chaperones, folding catalysts, and components of a unique degradation system known as ER-associated degradation (ERAD) (Ron and Walter 2007). UPR activation leads to translational arrest (reducing the influx of newly synthesized polypeptides into the ER), expansion of the ER membrane, and increased production of molecular chaperones and folding catalysts (increasing the protein folding capacity in the ER), and degradation of irreparable misfolded proteins by ERAD, thus re-establishing ER homeostasis and restoring ER function (Ron and Walter 2007). However, little is known about how UPR-induced proteins return to their pre-stress levels during the stress recovery phase. Several recent studies have suggested the involvement of a unique autophagy process, termed “recov-ER-phagy” upon severe ER stress in returning the expanded ER to its pre-stress size (Fumagalli et al. 2016; Loi et al. 2019).

The UPR is a highly conserved cellular stress pathway in eukaryotic organisms (Ron and Walter 2007; Hollien 2013; Howell 2021). Yeast is equipped with just 1 UPR branch consisting of an ER membrane-anchored IRE1 (inositol-requiring enzyme 1), a dual-functional Ser/Thr kinase and endoribonuclease that catalyzes a cytosolic splicing reaction to remove an intron from an mRNA to produce its translatable mRNA of HAC1 (homologous to ATF/CREB 1, a yeast basic-leucine zipper [bZIP] protein of 230 amino acids) (Hernandez-Elvira et al. 2018; Adams et al. 2019). Plants have 2 UPR branches, IRE1-bZIP60 (a plant equivalent of HAC1) and bZIP28/17 (Howell 2013). While bZIP17 requires 2 sequential proteolytic cleavages by site-1 protease (S1P) and site-2 protease (S2P) on the Golgi membrane, bZIP28 is cleaved only by S2P to release its transcriptionally-active fragments capable of translocation into the nucleus (Liu et al. 2007a; Tajima et al. 2008; Iwata et al. 2017a). The UPR systems in fission yeast, animals, and plants can also use IRE1, which can cleave certain mRNAs via regulated IRE1-dependent decay (RIDD) (Hollien and Weissman 2006), to reduce the influx of new proteins into the ER (Hollien et al. 2009; Kimmig et al. 2012; Mishiba et al. 2013).

In addition to decreasing the protein load and increasing the ER folding capacity, another consequence of UPR activation is enhanced ERAD efficiency. ERAD is a unique degradation mechanism that degrades ER-retained but irreparably misfolded proteins and consists of 4 interdependent steps: recognition/recruitment, retrotranslocation, ubiquitination, and proteasome-mediated proteolysis in the cytosol (Berner et al. 2018). The ERAD machinery is a multisubunit protein complex that builds around an ER membrane-anchored ubiquitin (E3) ligase. In yeast, at least 2 ERAD complexes are known: Hrd1 (HMG-reductase degradation 1) complex that degrades ERAD-L/M substrates with structural defects in their ER-luminal (L)/membrane (M) domains and Doa10 (Degradation of alpha2 10) complex that degrades ERAD-C clients carrying cytosol (C)-facing structural lesions (Zattas and Hochstrasser 2015). In Arabidopsis (*Arabidopsis thaliana*), there are at least 3 sets of ER membrane-anchored E3 ligases: AtHrd1a/b (2 homologs of the yeast Hrd1), Doa10a/b (2 homologs of the yeast Doa10), and RMA1-3 (Ring membrane-anchored 1-3) (Chen et al. 2020). The Arabidopsis Hrd1-containing ERAD complex contains several conserved proteins, AtHrd1a/b (Su et al. 2011), EBS6 (EMS-mutagenized bri1 suppressor 6)/AtOS9 (*A. thaliana* homolog of the mammalian osteosarcoma amplified-9) (Huttner et al. 2012; Su et al. 2012), EBS5/AtSel1A (*A. thaliana* homolog of the mammalian Sel1 for Suppressor of lin-12-like 1) (Liu et al. 2011; Su et al. 2011), an ER membrane-anchored ubiquitin conjugase UBC32 (U-box containing protein 32) (Cui et al. 2012), and 2 plant-specific components, EBS7 and PAWH1/2 (Protein Associated With Hrd1 1/2), which are important for regulating the stability of AtHrd1 (Liu et al. 2015; Lin et al. 2019).

TUNICAMYCIN-INDUCED 1 (TIN1) is an Arabidopsis protein that is rapidly induced by several chemical inducers of ER stress (Iwata et al. 2010a), such as tunicamycin (TM), a widely used inhibitor of the biosynthesis of the lipid-linked N-glycan precursor (Duksin and Mahoney 1982), dithiothreitol (DTT, a strong reducing agent that prevents the formation of disulfide bridges in newly synthesized and folded proteins), and azetidine-2-carboxylate (AZC, a proline analog whose incorporation into proteins causes protein misfolding [Trotter et al. 2001]). TIN1 is a plant-specific protein that lacks any known functional motif or domain and is highly expressed in pollen (Iwata et al. 2012). Previous studies using loss-of-function and overexpression approaches showed that TIN1 is involved in the formation of the pollen surface structure (Iwata et al. 2012, 2017b). However, little is known about TIN1's specific biochemical function and its post-stress recovery mechanism. Given TIN1's rapid induction by ER-stress inducers and its lack of any known domain or motif associated with protein folding and quality control, it was previously hypothesized that TIN1 might function as a protein cochaperone to assist protein folding, repair, and/or degradation (Iwata et al. 2010a). In this study, we found that TIN1, initially identified as a potential Hrd1-interacting protein in Arabidopsis, is an ER luminal protein that is detectable only in pollen, but not in vegetative tissues, under normal growth conditions. The loss-of-function *tin1* mutation had no significant impact on the Hrd1-mediated ERAD pathway and greatly reduced the thermotolerance during reproductive development. Our genetic and biochemical experiments indicated that ER-stress-induced TIN1 was rapidly degraded during the stress recovery phase via the Hrd1-containing ERAD complex in an asparagine-linked glycan (N-glycan) dependent manner. Interestingly, we found that neither BIP3 (immunoglobulin binding protein 3) nor ERdj3A (ER-localized DnaJ-domain protein 3A, also known as TMS1 for THERMOSENSITIVE MALE STERILE 1), 2 other well-known UPR-induced proteins (Noh et al. 2003; Shen and Hendershot 2005), exhibited rapid degradation upon removal of ER stress. Further investigation is needed to understand the biochemical basis for their differential post-ER-stress fates.

## Results

### TIN1 is a stress-induced ER luminal glycoprotein

A previous immunoprecipitation-mass spectrometry experiment, which identified PAWH1/2 as plant-specific components of the Arabidopsis ERAD complex (Lin et al. 2019), discovered TIN1 as an Hrd1-associated protein. *TIN1* was originally identified as an Arabidopsis gene that is highly induced by TM and was later found to be highly expressed in pollen (Iwata et al. 2010a, 2012). Consistent with earlier studies, the *TIN1* transcript was highly induced by several severe ER-stress inducers including TM, DTT, and AZC, which were revealed by a reverse transcription quantitative PCR (RT-qPCR) analysis and histochemical staining of young seedlings of a transgenic Arabidopsis line expressing a *pTIN1::GUS* (*β-glucuronidase*) reporter construct consisting of a *TIN1* promoter and the cDNA of the reporter β-glucuronidase (Supplementary Fig. S1A and B). Our RT-qPCR analyses also revealed that the *TIN1* transcript was induced by the plant stress hormone abscisic acid, mannitol (used widely as an osmotic stress inducer), and the 37 °C heat treatment (Supplementary Fig. S1, C to E). Interestingly, the salt treatment, which is known to activate an important UPR regulator bZIP17 (Liu et al. 2007b), had little impact on the *TIN1* transcript abundance (Supplementary Fig. S1F). We also analyzed the *TIN1* transcript abundance in TM-treated seedlings of Arabidopsis wild-type plants and several previously characterized UPR mutants. We discovered that the ER-stress-induced *TIN1* expression is largely mediated by the IRE1-bZIP60 branch of the Arabidopsis UPR mechanism. The TM induction of the *TIN1* transcript was greatly reduced in an *ire1a ire1b* double mutant, which lacks the 2 Arabidopsis homologs of the yeast IRE1, as well as in a *bzip60* mutant. In contrast, TIN1 induction was not affected in a *s2p* mutant, which lacks the Golgi-localized S2P involved in cleaving and activating bZIP17 and bZIP28, or in a *bzip28* mutant (Supplementary Fig. S1G).

**Figure 1.**
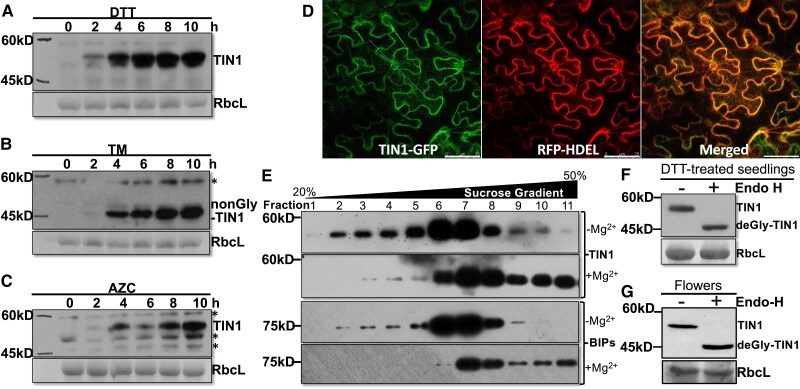
The TIN1 protein is an ER-localized protein that can be induced by ER stress. **A)** to **C)** Immunoblot analysis of the TIN1 accumulation in Arabidopsis seedlings treated with DTT **A)**, TM **B)**, and AZC **C)**. **D)** Confocal analysis of the subcellular localization of a transiently expressed TIN1-GFP fusion protein in tobacco leaf epidermal cells. Scale bar = 75 *μ*m. **E)** A sucrose gradient ultracentrifugation analysis of the endogenous TIN1 protein. **F)** and **G)** The Endo H analysis of the endogenous TIN1 protein in DTT-treated 2-wk-old seedlings or nontreated flowers of soil-grown mature plants. The wild-type seedlings used in **E** and **F** were pretreated with DTT for 4 h to increase the TIN1 abundance. In **A** to **C**, **F**, and **G**, the ponceau red-stained RbcL serves as the sample loading control. Asterisks in **B** and **C** indicate nonspecific cross-reacting bands, “non-Gly-TIN1” in **B** denotes the nonglycosylated form of TIN1, and “deGly-TIN1” in **F** and **G** indicates the deglycosylated form of TIN1 after the Endo H cleavage. The positions of molecular mass standards are shown on the left of images.

To study the impact of ER stress on the TIN1 protein level, we generated an anti-TIN1 antibody that detected the presence of TIN1 protein in DTT- or TM-treated wild-type seedlings and a TIN1-GFP (green fluorescent protein) fusion protein in a *p35S::TIN1-GFP* transgenic Arabidopsis line but not in stressed seedlings of a previously characterized T-DNA insertional *tin1-1* mutant (Iwata et al. 2012) (Supplementary Fig. S2A and B). As shown in Fig. 1, A to C, treatment with DTT, TM, or AZC resulted in significant accumulation of the TIN1 protein. Notably, the molecular weight of the TM-induced TIN1 protein was smaller than that of the DTT/AZC-induced TIN1 protein, likely due to the TM-induced inhibition of N-glycosylation. Consistent with the RT-qPCR results, the *pTIN1::GUS* histochemical staining (Supplementary Fig. S2C), and in an earlier gene expression analysis (Iwata et al. 2012), TIN1 was only detectable in the pollen of the Arabidopsis plants grown under normal conditions (Supplementary Fig. S2D).

In agreement with an earlier report (Iwata et al. 2010a), TIN1 is an ER-localized protein despite lacking the HDEL (His–Asp–Glu–Leu) ER retrieval motif at its C-terminus. As shown in Fig. 1D, a TIN1-GFP fusion protein exhibited an overlapping fluorescent pattern with HDEL-tagged red fluorescent protein (RFP-HDEL), a widely used ER marker protein (Liu and Dixon 2001), when they were coexpressed in tobacco (*Nicotiana benthamiana*) leaf epidermal cells. To eliminate the possibility that the overlapping ER localization patterns of TIN1-GFP and RFP-HDEL could be artifacts resulting from overexpression of an incorrectly folded GFP-fusion protein that carries a predicted N-terminal signal peptide in tobacco leaf epidermal cells (Fig. 1D) or in Arabidopsis protoplasts (Iwata et al. 2010a), we performed a sucrose gradient ultracentrifugation experiment in the presence or absence of Mg^2+^, which is known to be required for ribosome–ER association (Lord et al. 1973), with protein extracts of DTT-treated wild-type Arabidopsis seedlings. The DTT treatment was necessary to detect the TIN1 protein in Arabidopsis vegetative tissues. As shown in Fig. 1E, the addition of Mg^2+^ shifted the elution profile of the endogenous TIN1 protein towards higher density, similar to that of BIP1, a known ER-localized heat shock protein 70 (HSP70). Additional support for its ER localization was obtained by a simple biochemical assay utilizing endoglycosidase H (Endo H), which removes high mannose-type N-glycans of ER localized or retained glycoproteins but not Golgi-processed complex-type N-glycans of glycoproteins that traffic through the Golgi apparatus (Tarentino et al. 1974). As shown in Fig. 1, F and G, the endogenous TIN1 proteins of the DTT-treated wild-type Arabidopsis seedlings and nontreated flowers were sensitive to Endo H. In line with the predictions of a signal peptide and protein topology of TIN1, extraction of the microsomal pellets of the wild-type Arabidopsis seedlings with 0.1 m NaCl, 0.1 m Na_2_CO_3_, 1% Triton X-100, and 1% NP-40 indicated that TIN1 is an ER luminal protein (Supplementary Fig. S3). Taken together, our experiments confirmed that the endogenous TIN1 protein is an ER-localized soluble glycoprotein.

### TIN1 and its homolog play no significant role in ERAD of mutant receptor-like kinases bri1-5 or bri1-9

**Figure 2.**
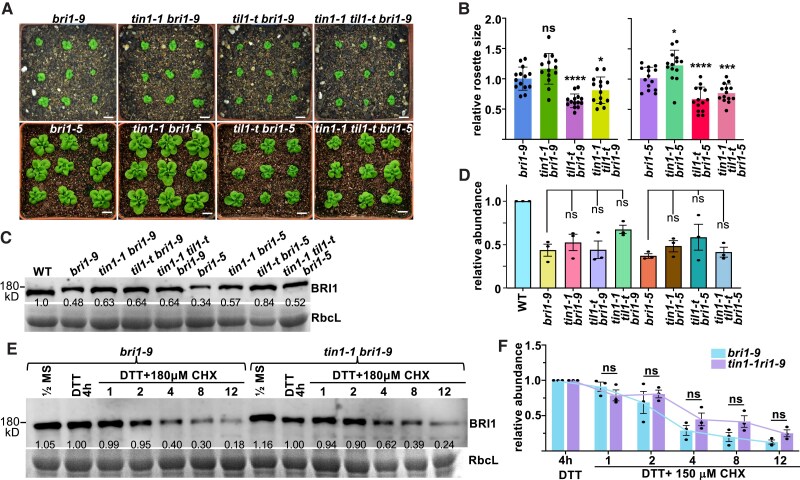
TIN1 and TIL1 play no significant role in ERAD of bri1-5 and bri1-9. **A)** Pictures of 2-wk-old soil-grown plants. Scale bar = 10 mm. **B)** A bar graph of average rosette sizes of Arabidopsis seedlings of the indicated genotypes. ImageJ was used to measure the rosette sizes of double or triple mutants relative to the parental *bri1* mutants (set to 1). Each bar represents the mean rosette size of an indicated mutant, with individual data points shown and error bars indicating ± SD (*n* = 14). **C)** Immunoblot analysis of BRI1 abundance in 2-wk-old seedlings of various genotypes with the ponceau red-stained RbcL band serving as the loading control. The numbers are the ImageJ-generated anti-BRI1 signal intensity of various mutants relative to the wild-type after normalization with the RbcL signals. **D)** A bar graph showing the average anti-BRI1 signal intensity from 3 independent immunoblots of the indicated mutants. Individual data points were plotted on each bar with error bars denoting ± SD (*n* = 3). **E)** Immunoblot analysis of the bri1-9 stability of the *bri1-9* and *tin1-1 bri1-9* mutants with ponceau red-stained RbcL band serving as the loading control. Two-week-old seedlings were pretreated with H_2_O (mock) or 2 mm DTT for 4 h and transferred into liquid 1/2 mS medium containing 2 mm DTT and 180 *μ*M CHX for incubation. Equal amounts of seedlings were collected at the indicated time points for the extraction of total proteins, which were separated by SDS-PAGE and analyzed by immunoblotting with the BRI1 antibody. The numbers represent ImageJ-quantified anti-BRI1 signal intensity of the DTT/CHX-treated seedlings collected at indicated time points, normalized to the RbcL loading control, relative to the 2 mm DTT-treated sample (for 4 h), which was set to 1. **F)** A bar graph depicting the average relative anti-BRI1 signal intensity in chemical-treated seedlings of *bri1-9* and *tin1 bri1-9*. Individual data points of 3 experiments were plotted on each bar with error bars indicating ± SD. In **C** and **E**, the positions of molecular mass standards are shown to the left of the images. For **B** and **D**, statistical significance was determined using Student's *t*-test, while for **F**, 2-way ANOVA analysis using SPSS was performed: * *P* < 0.05; ** *P* < 0.01; *** *P* < 0.001; **** *P* < 0.0001, ns indicates no significance difference.

Because of its coimmunoprecipitation with Hrd1 and its ER localization, we hypothesized that TIN1 might be involved in the Hrd1-mediated ERAD pathway, which degrades 2 ER-retained mutant variants of the brassinosteroid (BR) receptor BRASSINOSTEROID-INSENSITIVE 1 (BRI1), including bri1-5 and bri1-9 (Chen et al. 2020). Our previous studies showed that mutations in components or regulators of the Hrd1-containing ERAD complex greatly stabilized the 2 mutant bri1 receptors and partially rescued the growth phenotypes of the corresponding dwarf mutants due to leakage of ER-accumulated bri1-5 or bri1-9 proteins to the plasma membrane where the mutant BRI1 receptors activate the BR signaling pathway to promote plant growth (Su et al. 2011, 2012; Liu et al. 2015; Lin et al. 2019). If TIN1 were involved in ERAD, a loss-of-function *tin1* mutation would inhibit degradation of the 2 mutant bri1 receptors and suppress their dwarf phenotype. To test this hypothesis, we crossed *tin1-1* with *bri1-5* and *bri1-9* to generate the *tin1-1 bri1-5* and *tin1-1 bri1-9* double mutants. While *tin1-1* had no significant impact on the small rosette phenotype of *bri1-9,* it significantly enlarged the rosette size of *bri1-5*, which could be caused by ecotype difference with *bri1-5* in Ws-2 (Wassilewskija-2) ecotype and *tin1-1* in Col (Columbia) ecotype (Fig. 2, A and B; Supplementary Fig. S4A). However, repeated immunoblot analyses detected no significant effect of the *tin1-1* mutation on the protein abundance of not only bri1-9 but also bri1-5 (Fig. 2, C and D; Supplementary Fig. S4B).

We suspected that the weak or no impact of *tin1-1* on the dwarfism of the 2 *bri1* mutants and the bri1 protein abundance could be due to the low abundance of TIN1 protein, which is below the detection limit of our anti-TIN1 antibody, in the vegetative tissues (Supplementary Fig. S2). To further investigate a potential ERAD role of TIN1, we performed a cycloheximide (CHX)-chasing experiment, which was previously used to analyze the stability of the mutant bri1 proteins, as CHX is a well-known inhibitor of protein synthesis (Hong et al. 2009, 2012). We pretreated seedlings of *bri1-9* and *tin1-1 bri1-9* with 2 mm DTT for 4 h to induce TIN1 accumulation in vegetative tissues and added 2 mm DTT to the CHX assay solution. Repeated immunoblot analyses revealed no significant difference in the stability of the mutant bri1-9 receptor between *bri1-9* and *tin1-1 bri1-9* mutants (Fig. 2, E and F; Supplementary Fig. S4C). Taken together, these results indicated that the *tin1-1* mutation had no significant impact on the ERAD of the 2 mutant BR receptors despite its suppressive effect on the rosette size of the *bri1-5* mutant.

Alternatively, the lack of a significant impact of the *tin1-1* mutation on the degradation of bri1-5 and bri1-9 or the *bri1-9* dwarfism could stem from functional redundancy between TIN1 and a potential TIN1-like protein (At1g47310, named hereinafter TIL1 for TIN1-Like1). TIL1 is a 395-amino acid polypeptide exhibiting 22%/58% sequence identity/similarity with TIN1 (Supplementary Fig. S5A), and the presence of the TIN1-TIL1 pair is widely detected in the sequenced genomes of seed plants (Supplementary Fig. S5B). Similar to TIN1, TIL1 carries a predicted N-terminal signal peptide (Supplementary Fig. S6) and is likely localized in the ER as revealed by confocal microscopy of a TIL1-GFP fusion protein transiently expressed in tobacco leaves (Supplementary Fig. S7A) and sucrose-gradient ultracentrifugation analyses of the TIL1-GFP fusion protein expressed in transgenic Arabidopsis plants (Supplementary Fig. S7B). It is important to note that *TIL1* is a constitutively expressed gene insensitive to TM treatment (Supplementary Fig. S7C and D). Consistent with its ER localization, the TIL1-GFP fusion protein was sensitive to Endo H cleavage (Supplementary Fig. S7E).

To test the potential functional redundancy of TIN1 and TIL1 in an Arabidopsis ERAD process, we obtained a T-DNA insertional mutant *til1* from ABRC (*SAIL_165_E10* containing a T-DNA insertion in the 1st exon, also in Col ecotype), which was morphologically indistinguishable from the *tin1-1* mutant or its wild-type control (Supplementary Fig. S8), and generated *til1 bri1-9*, *til1 bri1-5*, *tin1 til1 bri1-9*, and *tin1 til1 bri1-5* mutants. Interestingly, we found that the *til1* mutation, as well as the *tin1 til1* double mutation, actually enhanced but did not suppress the small rosette phenotype of the 2 *bri1* mutants (Fig. 2, A and B; Supplementary Fig. S4A). However, repeated immunoblot analyses detected no statistically significant difference in the protein abundance of mutant bri1-5 and bri1-9 receptors between the 2 single *bri1* mutants and their corresponding *til1 bri1* double or *tin1 til1 bri1* triple mutants (Fig. 2, C and D; Supplementary Fig. S4B). We also overexpressed the fusion protein of TIN1-GFP or TIL1-GFP in the *bri1-9* mutant but discovered that neither fusion protein had a significant impact on the growth phenotype of the dwarf mutant (Supplementary Fig. S9A and B). Based on these statistical analyses and what was known for the components or regulators of the Hrd1-mediated ERAD pathway, we concluded that neither TIN1 nor TIL1 has a regulatory function for the degradation of the 2 mutant bri1 receptors. It should be emphasized that our results do not exclude the possibility that TIN1 and/or TIL1 might be involved in an ERAD pathway to degrade yet unknown ERAD substrates.

### TIN1 contributes to thermotolerance during reproductive development

**Figure 3.**
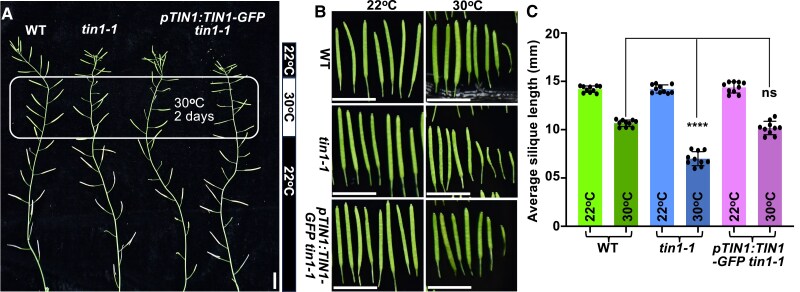
The *tin1-1* mutant exhibits reduced fertility at 30 °C. **A)** Shown here are representative silique pictures of the mature Arabidopsis plants of the indicated genotypes. Flowering plants with many developed siliques grown at 22 °C were transferred into a 30 °C growth chamber for 2 d and moved back to 22 °C. The rounded rectangular region indicates silique developed at 30 °C, and the bar on the right shows the temperature shift scheme. Scale bar = 10 mm. **B)** Representative images of siliques harvested from the rectangular region indicated in **A**, compared with siliques collected from the corresponding regions of plants of the same genotypes continuously grown at 22 °C. Scale bar = 10 mm. **C)** Quantitative analysis of average lengths of siliques developed at 22 or 30 °C. Green mature siliques were harvested, photographed, and digitally analyzed using the NIH ImageJ for measurement of silique lengths. Approximately 80 siliques (10 plants with ∼8 siliques/plant) of each genotype were analyzed to obtain the average silique length, with each data point indicating the average length of ∼8 siliques of each analyzed plant. Error bars indicate ± SD (*n* = 10). The statistical significance was performed using the Student's *t*-test; ****, *P* < 0.0001; ns, no statistical significance.

Earlier studies showed that the pollen grains of the *tin1-1* mutant and *TIN1*-overexpression transgenic Arabidopsis lines exhibit defective surface structure (Iwata et al. 2012, 2017b). However, despite repeated electron microscopic observations, we did not detect any obvious difference in the surface of pollen grains between the *tin1-1* mutant or the *TIN1-GFP* overexpression line and the wild-type Arabidopsis plants (Supplementary Fig. S10). We suspected that such a phenotypic inconsistency could be caused by growth or experimental conditions. It is well known that many of the UPR genes are constitutively expressed in pollen and play important roles in pollen development and thermotolerance (Singh et al. 2021). We therefore investigated whether the *tin1-1* mutant might exhibit a thermo-sensitive phenotype during reproductive development. We transferred 7-wk-old flowering Arabidopsis plants grown in a 22 °C growth room into a 30 °C growth chamber for a 2-d heat treatment and moved them back into the 22 °C growth room for continued growth and phenotypic analysis. As shown in Fig. 3, while the temperature shift had little impact on the wild-type plants, it markedly reduced the fertility and the average silique length of the *tin1-1* mutant. Importantly, such thermo-sensitivity phenotypes were rescued by a *pTIN1::TIN1-GFP* transgene. These results also prompted us to examine the surface phenotype of pollens formed during the 2-d growth at 30 °C. As shown in Supplementary Fig. S11, the 2-d heat treatment indeed caused observable pollen phenotypes in the *tin1-1* mutant but not in the wild-type or the complemented *pTIN1::TIN1-GFP tin1-1* line. In addition to the electron-dense whitish deposits on the pollen surface (indicated by white arrowheads in Supplementary Fig. S11), some of the pollens showed irregular, deformed, or collapsed structures (indicated by white stars in Supplementary Fig. S11). It is worth noting that the *til1-t* mutation did not enhance the *tin1-1*'s pollen phenotypes (Supplementary Fig. S11F and G). Thus, we concluded that TIN1 is an important thermotolerance factor during reproductive development.

### TIN1 is rapidly degraded, while TIL is a stable protein

**Figure 4.**
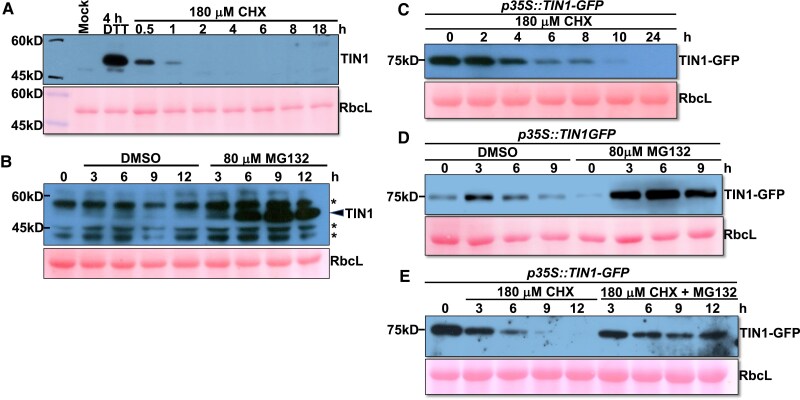
TIN1 is rapidly degraded via a proteasome-mediated process. **A)** Immunoblot analysis of the TIN1 stability in seedlings treated with DTT (for 4 h) followed by CHX for varying durations. The 2-wk-old seedlings were carefully transferred from 1/2 mS agar medium into the liquid 1/2 MS medium supplement without (mock) or with 2 mm DTT for 4 h and transferred again into fresh liquid 1/2 medium for treatment with 180 *μ*M CHX for different durations. **B)** Immunoblot analysis of the TIN1 protein abundance in seedlings treated with DMSO or MG132. Asterisks indicate nonspecific cross-reacting bands. **C)** Immunoblot analysis of the protein stability of a TIN1-GFP fusion protein in *p35S::TIN1-GFP* transgenic seedlings treated with 180 *μ*M CHX for different durations. **D)** and **E)**. Immunoblot analysis of the TIN1-GFP protein abundance in *p35S::TIN1-GFP* seedlings treated with DMSO or MG132 in the presence or absence of CHX. For all the immunoblots, the ponceau red-stained RbcL bands were used for the loading control, and the positions of molecular mass standards are shown on the left.

We also investigated the possibility that TIN1 is a potential ERAD substrate. We performed a cycloheximide (CHX)-chasing experiment to analyze the stability of the DTT-induced TIN1 protein. Because the TIN1 abundance in vegetative tissues is often below the detection limit of our anti-TIN1 antibody (Supplementary Fig. S2), a 4-h pretreatment with DTT was necessary to permit easy TIN1 detection for the CHX-chasing experiment (Supplementary Fig. S2A). As shown in Fig. 4A, The DTT-induced TIN1 was rapidly degraded with its half-life being ∼30 min. It is important to note that following the DTT removal, the mRNA levels of several UPR genes, including *TIN1*, were gradually reduced during the 24-h incubation period (Supplementary Fig. S12). Additional support for TIN1 being a very unstable protein came from our analysis of the TIN1 abundance of Arabidopsis seedlings treated with dimethyl sulfoxide (DMSO) or MG132, a widely used inhibitor of the proteasome. As shown in Fig. 4B, the DMSO-treated seedlings accumulated no detectable or extremely low amounts of TIN1 protein, whereas the MG132 treatment resulted in strong accumulation of TIN1. We also tested the protein stability of AZC-induced TIN1 and found that the AZC-induced TIN1 protein was also degraded upon AZC removal (Supplementary Fig. S13). Because both DTT and AZC can cause TIN1 misfolding or disrupt TIN1 interaction with other ER-localized proteins to reduce its protein stability, we also analyzed the stability of a TIN1-GFP fusion protein in a *p35S::TIN1-GFP* transgenic line using the CHX-chasing approach and found that TIN1-GFP was also an unstable protein, with a 6-fold longer half-life than the DTT-induced endogenous TIN1 protein (Fig. 4C). Similarly, treatment with MG132 also increased the stability of the TIN1-GFP fusion protein in the absence or presence of CHX (Fig. 4, D and E). It is worth noting that the stability of TIN1-GFP is stronger than that of the DTT/AZC-induced endogenous TIN1, likely caused by the strong *p35S* promoter or the C-terminal GFP tag that might interfere with TIN1 degradation. Taken together, these results demonstrated that TIN1 is a highly unstable protein under ER-stress conditions or when overexpressed, which is likely permanently degraded by the cytosolic proteasome in vegetative tissues. By contrast, TIL1 is a very stable protein. A CHX-chasing experiment revealed a little change in the abundance of TIL1-GFP in *p35S::TIL1-GFP* transgenic seedlings (Supplementary Fig. S14).

### TIN1 degradation relies on its N-glycans

**Figure 5.**
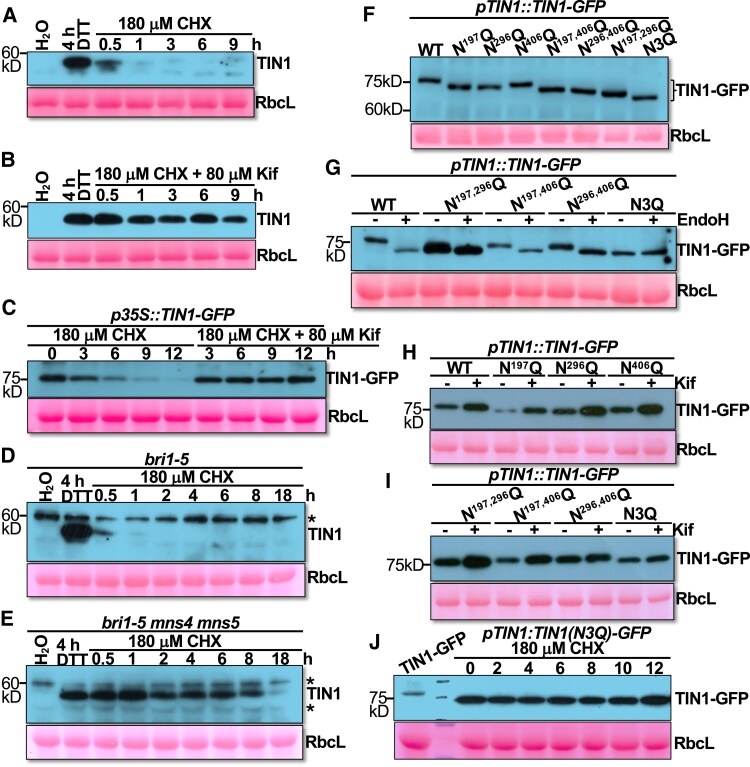
TIN1 degradation relies on its N-glycans. **A)** and **B)** Immunoblot analysis of the TIN1 protein abundance in 2-wk-old seedlings treated with H_2_O or DTT for 4 h followed by CHX (for varying durations) in the absence **A)** or presence **B)** of Kif. **C)** Immunoblot analysis of the TIN1-GFP abundance in 2-wk-old *p35S::TIN1-GFP* transgenic seedlings treated with CHX for different durations in the presence or absence of Kif. **D)** and **E)** Immunoblot analysis of the TIN1 protein level in 2-wk-old seedlings of *bri1-5*  **D)** or *bri1-5 mns4 mns5* triple mutant **E)**, which were treated with DTT for 4 h followed by treatment with 180 *μ*M CHX for varying durations. **F)** Analysis of the Asn-Gln mutations on the electromobility of the TIN1-GFP protein on SDS-PAGE. **G)** The Endo H sensitivity of various Asn-Gln mutated TIN1-GFP proteins. **H)** and **I)** The Kif sensitivity of various Asn-Gln-mutated TIN1-GFP proteins. **J)** The immunoblot analysis of the TIN1(N3Q)-GFP protein abundance in 2-wk-old *p35S::TIN1(N3Q)-GFP* seedlings treated with 180 *μ*M CHX for varying durations. In (A to J), the ponceau red-stained RbcL serves as the loading control, with the positions of molecular mass standards shown on the left. In **D** and **E**, asterisks indicate nonspecific cross-reacting bands.

Because TIN1 is an N-glycosylated protein (Fig. 1F), we investigated whether its rapid degradation upon removal of an ER stressor depends on its N-glycans. We first treated the wild-type Arabidopsis seedlings with DTT to induce TIN1 accumulation, followed by treatment with CHX in the presence or absence of kifunensine (Kif), a well-known inhibitor of ERAD by blocking α1,2-mannosidases that generate the conserved N-glycan ERAD signal carrying a terminal α1,6-mannose residue (Elbein et al. 1990). As shown in Fig. 5, A and B, TIN1 remained detectable 9 h after CHX addition in the presence of Kif, whereas TIN1 abundance was markedly reduced 1 h after CHX addition in the absence of Kif, suggesting that TIN1 degradation is likely N-glycan-dependent. A similar experiment performed with seedlings of the *p35S::TIN1-GFP* transgenic line also revealed that Kif effectively prevented the CHX-triggered disappearance of TIN1-GFP (Fig. 5C).

A further support for the N-glycan-dependent degradation of TIN1 came from our analysis of TIN1 stability in an Arabidopsis double mutant lacking the 2 Arabidopsis homologs of the mammalian ER-degradation enhancing α-mannosidase-like proteins (EDEMs), α-mannosidase 4 (MNS4) and MNS5. Earlier studies showed that MNS4 and MNS5 function redundantly in degrading the ER-retained bri1-5 (Huttner et al. 2014; Sun et al. 2022; Zhang et al. 2022). As shown in Fig. 5, D and E, a CHX-chasing experiment with *bri1-5* mutant seedlings revealed a rapid degradation of the DTT-induced TIN1, whereas a similar CHX-chasing experiment performed with seedlings of the *mns4 mns5 bri1-5* triple mutant showed that the DTT-induced TIN1 protein remained stable for at least 8 h after the CHX treatment. We conclude that TIN1 degradation is largely dependent on its N-glycans.

### Both N^296^ and N^406^ are important for TIN1 degradation

Sequence analysis indicated that TIN1 has 3 potential N-glycosylation sites: N^197^ (N for asparagine), N^296^, and N^406^ (Supplementary Fig. S5A). To determine whether or not all 3 predicted N-glycan sites are indeed glycosylated and involved in TIN1 degradation, we generated Arabidopsis transgenic lines expressing one of the 7 TIN1-GFP fusion proteins lacking 1 (N^197^Q, N^296^Q, N^406^Q; Q for glutamine), 2 (N^197,296^Q, N^197,406^Q, N^296,406^Q), or all 3 (N^197,296,406^Q [N3Q]) potential N-glycosylation sites and subsequently analyzed the impact of these 7 mutations on the mobility and Endo H or Kif sensitivity of mutant TIN1-GFP fusion proteins. Representative transgenic lines were used for the biochemical assays. As shown in Fig. 5F, all 7 mutations resulted in faster mobility of the TIN1-GFP fusion proteins on sodium dodecyl sulfate-polyacrylamide gel electrophoresis (SDS-PAGE). It is interesting to note that the 3 single N-Q mutant TIN1-GFP fusion proteins exhibited different mobility on the SDS-PAGE gel, with TIN1^N406Q^-GFP running a bit slower than TIN1^N197Q^-GFP and TIN1^N296Q^-GFP, which may result from the altered amino acid context. Consistent with this observation, the TIN1^N197,406Q^-GFP and TIN1^N296,406Q^-GFP bands moved a bit slower than the TIN1^N197,296Q^-GFP band on the SDS-PAGE gel. In line with the mobility difference of the 7 mutant TIN1-GFP fusion proteins, the Endo H treatment showed that while the Endo H digestion caused no change in the mobility of the TIN1^N197, 296, 406Q^-GFP (TIN1[N3Q]-GFP), it did result in mobility change of the 3 double N-Q mutant TIN1-GFP fusion proteins on SDS-PAGE gel (Fig. 5G). Together, these experiments confirmed that TIN1 is N-glycosylated at the 3 predicted N residues.

We also treated those transgenic seedlings with 25 *μ*M Kif for 6 h and analyzed the TIN1 protein abundance by immunoblotting. As shown in Fig. 5H, the Kif treatment seemed to have a stronger impact on TIN1^N197Q^-GFP than TIN1^N296Q^-GFP or TIN1^N406Q^-GFP. Consistently, Kif increased the protein abundance of TIN1^N197,296Q^-GFP and TIN1^N197,406Q^-GFP proteins but had a much weaker effect on the protein abundance of TIN1^N296,406Q^-GFP and TIN1(N3Q)-GFP (Fig. 5I). These results suggest that N^296^ and N^406^ have a stronger impact on TIN1 degradation than N^197^. More importantly, a CHX-chasing experiment with seedlings of the *p35S:TIN1(N3Q)-GFP* transgenic line indicated that simultaneous elimination of all 3 N-glycosylation sites effectively blocked the TIN1 degradation (Fig. 5J). These results not only confirmed the N-glycan dependence of TIN1 degradation but also revealed potential functional differences among the 3 glycosylated N residues. It is interesting to note that our sequence analysis showed that the 3 predicted N-glycosylation sites are highly conserved among the plant TIN1 homologs, with the N^197^ residue being the least conserved and only present in TIN1 homologs of angiosperms (Iwata et al. 2010a) (Supplementary Fig. S15).

### TIN1 degradation requires both EBS6 and EBS5

**Figure 6.**
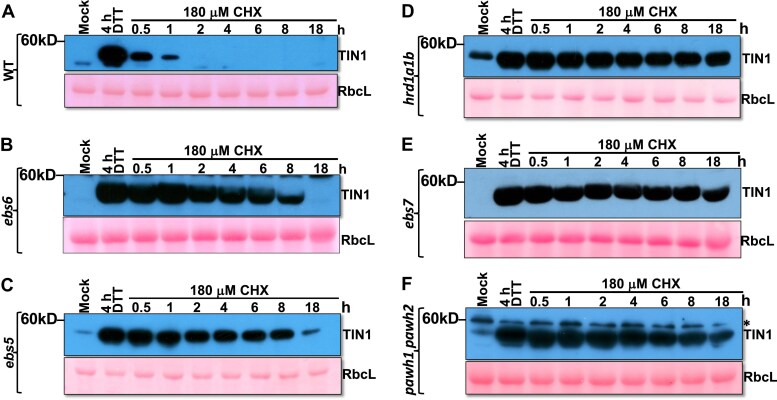
TIN1 degradation requires core components of the Arabidopsis Hrd1 complex. **A)** to **F)** Immunoblot analysis of the TIN1 protein abundance in 10-d-old seedlings of indicated genotypes, including wild-type, *ebs5*, *ebs6*, *hrd1a1b*, *ebs7*, and *pawh1 pawh2*, which were treated with H_2_O (mock) or DTT for 4 h followed by treatment with 180 *μ*M CHX for varying durations. The ponceau red-stained RbcL serves as the loading control in each immunoblot assay, with the positions of molecular mass standards shown to the left of images. The asterisk in F indicates the nonspecific cross-reacting band.

Our results of the Kif treatment, the Arabidopsis *mns4 mns5 bri1-5* mutant, and the Arabidopsis transgenic lines expressing loss-of-glycosylation mutant TIN1-GFP fusion proteins strongly suggested that TIN1 degradation likely requires an ERAD N-glycan signal with an exposed α1,6-mannose residue (Hong et al. 2009, 2012). Previous studies showed that such an ERAD signal is recognized in Arabidopsis by a highly conserved Arabidopsis protein EBS6 (Su et al. 2012), which is also known as AtOS9 for its sequence similarity to the mammalian OS9 and the yeast yOS9 (yeast OS9 homolog) (Huttner et al. 2012). We thus predicted that EBS6 is required for the TIN1 degradation. To test our prediction, we performed a CHX-chasing experiment with seedlings of a previously described *ebs6-2* mutant and its corresponding wild-type (Su et al. 2012) and found that the *ebs6-2* mutation greatly stabilized the DTT-induced TIN1 protein compared to that of the wild-type seedlings (Fig. 6, A and B). Intriguingly, the *ebs6-2* mutation did not completely block the TIN1 degradation as TIN1 was not detectable in *ebs6-2* seedlings 18 h after the CHX treatment (Fig. 6B). This finding seemed to be consistent with the result of the CHX-chasing experiment of the *bri1-5 mns4 mns5* triple mutant that had only a residual amount of TIN1 protein 18 h after the CHX addition (Fig. 5E).

A similar CHX study was also performed with seedlings of another Arabidopsis ERAD mutant, *ebs5*, which carries a T-DNA insertional mutation of EBS5 (Su et al. 2011), the Arabidopsis homolog of the yeast Hrd3/mammalian Sel1L. Both Hrd3 and Sel1L are thought to be an ERAD substrate recruitment factor that specifically recognizes and binds exposed hydrophobic regions in a misfolded protein. It was hypothesized that the hydrophobicity-perceiving Hrd3/Sel1L interacts with the N-glycan-recognizing yOS9/OS9 to recruit a terminally misfolded glycoprotein to the ER membrane-anchored E3 ligase for retrotranslocation and ubiquitination (Liu et al. 2011; Su et al. 2011), thus constituting a double-checking mechanism to increase the specificity of the ERAD system that only degrades terminally misfolded proteins but not folding intermediates. It is also important to note that the EBS5-EBS6 interaction is required to retain EBS6, which lacks the HDEL ER retention signal, in the ER, and that *ebs5* mutations lead to EBS6 degradation (Su et al. 2012). Similar to what was found in the *ebs6-2* mutant, the introduced *ebs5* mutation markedly inhibited but did not completely eliminate the TIN1 degradation (Fig. 6C). Together, these biochemical experiments demonstrated that degradation of the DTT-induced TIN1 requires the 2 interacting recruitment factors known to bring a committed glycosylated ERAD client to the ER membrane-anchored ERAD machinery.

### TIN1 degradation is mediated by the EBS7-PAWH-Hrd1 complex

Because TIN1 is an ER luminal HDEL-lacking glycoprotein that requires EBS5 and EBS6 for its degradation, we suspected that TIN1 likely uses the ER membrane-anchored E3 ligase Hrd1 complex for its retrotranslocation and ubiquitination. Our previous studies demonstrated the redundant function of the 2 Arabidopsis homologs of the yeast Hrd1, AtHrd1a and AtHrd1b, in ERAD of bri1-5 and bri1-9 (Su et al. 2011). To investigate a potential role of AtHrd1a/1b in TIN1 degradation, we first examined the TIN1 abundance in several known ERAD mutants, including an Arabidopsis *hrd1a hrd1b* double mutant, and found that TIN1 was easily detected in the *hrd1a hrd1b* double mutant that was not treated with an ER-stress-inducing chemical (Supplementary Fig. S16A). We believed that this was likely caused by the *hrd1a hrd1b*-blocked degradation of the TIN1 protein rather than by the *hrd1a hrd1b*-triggered increase in *TIN1* transcript (Supplementary Fig. S16B). By comparison, the heat treatment caused a much greater increase (∼40 fold [Supplementary Fig. S1E]) in *TIN1* transcript abundance than the *hrd1a hrd1b* double mutation (∼4 fold [Supplementary Fig. S16B]), yet the heat-treated seedlings failed to accumulate a detectable amount of TIN1 protein (Supplementary Fig. S17A). A further support for our conclusion was provided by the CHX-chasing experiments that revealed a very stable TIN1 protein in CHX-treated or heat-stressed *hrd1a hrd1b* mutant seedlings (Fig. 6D; Supplementary Fig. S17B) and a very stable TIN1-GFP fusion protein in *p35S::TIN1-GFP hrd1a hrd1b* transgenic line (Supplementary Fig. S17C).

Our recent studies discovered that the protein stability and/or activity of the Arabidopsis Hrd1 is regulated by 2 plant-specific components of the Arabidopsis Hrd1-containing ERAD machinery, EBS7 and PAWH1/2 (Liu et al. 2015; Lin et al. 2019). Consistently, our immunoblot analysis of the TIN1 abundance in *ebs7* and *pawh1 pawh2* mutants showed increased TIN1 abundance in the 2 ERAD mutants (Supplementary Fig. S16A). Interestingly, the *ebs6* mutant accumulated no detectable amount of TIN1 protein compared to other ERAD mutants (Supplementary Fig. S16A), which is likely caused by a potential direct TIN1-Hrd1 interaction in the ER lumen to compensate for the loss of the TIN1-EBS6 binding. The increased TIN1 abundance in the *ebs5* mutant at 18 h after CHX treatment in comparison to the *ebs6* mutant (Fig. 6, B and C) and high accumulation of TIN1 protein in the nonstressed *ebs5* mutant (Supplementary Fig. S16A) might be caused by a combination of no EBS5 and significantly reduced abundance of the ER-retained EBS6 in the *ebs5* mutant (Su et al. 2012). We have previously shown that neither *ebs5* nor *ebs6* mutation had an obvious impact on the protein abundance of EBS7, PAWH1/2, or Hrd1 (Lin et al. 2019). We also performed the CHX-chasing experiment with seedlings of *ebs7*-*1* and *pawh1 pawh2* mutants that were known to be defective in ERAD of 2 mutant bri1 proteins (Liu et al. 2015; Lin et al. 2019). As shown in Fig. 6, E and F, both *ebs7* and *pawh1 pawh2* mutations greatly stabilized the DTT-induced TIN1 protein. Similar CHX-chasing experiments were conducted with AZC-treated seedlings of *ebs7* and *hrd1a hrd1b* mutants, which revealed that both the *ebs7* and *hrd1a hrd1b* mutations almost completely blocked degradation of the AZC-induced TIN1 (Supplementary Fig. S18A and B). Taken together, these biochemical analyses indicated that the ER-stress-induced TIN1 is degraded by the EBS7-PAWH1/2-Hrd1 ERAD machinery.

### BIP3 and ERdj3A are highly stable proteins

**Figure 7.**
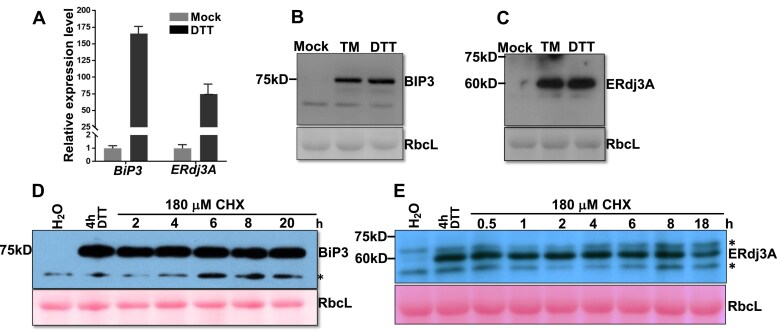
The UPR-induced BIP3 and ERdj3A are very stable proteins. **A)** A bar graph showing the transcript levels of *BIP3* and *ERdj3A* in DTT-treated seedlings in comparison to the mock-treated seedlings. The value of each represents the average result of 3 biological replicates, and error bars indicate ± SD. **B)** and **C)** Immunoblot analysis of the protein abundance of BIP3 **B)** and ERdj3A **C)** in wild-type seedlings treated with or without TM or DTT. **D)** and **E)** Immunoblot analysis of the protein abundance of BIP3 **D)** and ERdj3A **E)** in seedlings treated with DTT (for 4 h) followed by 180 *μ*M CHX (for varying durations). In **A** to **E**, mock treatment indicates that seedlings were incubated in liquid 1/2 MS containing no ER-stress-inducing chemical. In **B** to **E**, the ponceau red-stained RbcL was used as the loading control, and the asterisks indicate nonspecific cross-reacting bands. The positions of molecular mass standards are shown to the left of blot images.

Our finding that a UPR-induced protein is rapidly degraded via ERAD prompted us to examine the stability of 2 other UPR-induced Arabidopsis proteins upon resolution of the ER stress. Previous studies showed that *BIP3* and *ERdj3A* were rapidly and greatly induced by several ER-stress inducers (Noh et al. 2003; Iwata and Koizumi 2005; Yamamoto et al. 2008; Yang et al. 2009; Iwata et al. 2010b) and that these 2 genes were known to be coexpressed with *TIN1* (Supplementary Fig. S19). BIP3 is one of the 3 ER-localized HSP70s in Arabidopsis (Noh et al. 2003), while ERdj3A/TMS1 is a member of the ER-localized HSP40s functioning as co-chaperones of HSP70s and interacts with BIP3 (Yamamoto et al. 2008; Ma et al. 2015). In line with previous results, the transcript abundance and protein levels of BIP3 and ERdj3A/TMS1 were greatly upregulated by DTT and TM (Fig. 7, A to C). To investigate if BIP3 and ERdj3A were also rapidly degraded upon removal of DTT, we performed similar DTT-CHX time-course analyses for BIP3 and ERdj3A. As shown in Fig. 7, D and E, both BIP3 and ERdj3A remained very stable 24 h after CHX treatment of the DTT-pretreated seedlings. Thus, the rapid degradation via ERAD upon removal of ER stress is not a common recovery mechanism for UPR-induced proteins but likely a unique property of TIN1 or only applies to a subset of UPR-induced proteins, raising the interesting question of what unique features make TIN1 a target of the Arabidopsis ERAD machinery, compared to BIP3, ERdj3A, or its stable homolog TIL1.

### Discussion

In this study, we have shown that the endogenous TIN1 is an ER luminal protein that is rapidly degraded after its induction by UPR. Although an earlier study, which used protoplasts expressing a TIN1-GFP fusion protein, suggested that TIN1 was an ER-localized protein (Iwata et al. 2010a), it remains unknown whether the endogenous TIN1 is also retained in the ER, as the predicted TIN1 protein contains the N-terminal signal peptide but lacks a recognizable ER retention/retrieval motif. Besides, the C-terminal fusion of a GFP tag might prevent its packaging into trafficking vesicles for moving into the Golgi body, similar to what was previously reported (Silva-Alvim et al. 2018). Because we have successfully generated a TIN1-specific antibody, we performed 2 biochemical assays to study the subcellular localization of the endogenous TIN1 protein in addition to confocal microscopic analysis of a TIN1-GFP fusion protein that was transiently expressed in tobacco leaf epidermal cells. The TIN1 protein was predicted to contain 3 N-glycosylation sites that were confirmed in this study by an Endo H assay of the endogenous TIN1 protein and analyses of Endo H and Kif sensitivity of 7 transgenically expressed TIN1-GFP fusion proteins lacking 1, 2, or all 3 predicted N-glycosylation sites. The Endo H assay was also used to confirm the ER localization of the endogenous TIN1 protein in DTT-treated seedlings and the nontreated flowers of Arabidopsis because its N-glycans could easily be deglycosylated by Endo H, which cleaves high mannose-type N-glycans of ER-localized or retained glycoproteins but not Golgi-processed complex-type N-glycans. TIN1's ER localization was further confirmed by sucrose gradient ultracentrifugation in the presence or absence of Mg^2+^ that is required for the association of ribosomes with the ER (Lord et al. 1973). Consistent with the signal peptide prediction at SignalP6.0 (https://dtu.biolib.com/SignalP-6) (Teufel et al. 2022) and Aramemnon (https://aramemnon.botanik.uni-koeln.de/) (Schwacke and Flügge 2018) (Supplementary Fig. S3), our resuspension of the microsomal pellets of the wild-type Arabidopsis seedlings using different buffer conditions showed that TIN1 is an ER luminal protein even though it lacks the HDEL ER-retrieval motif. We suspect that TIN1 likely interacts with an ER-localized protein to stay in the folding compartment. Previous studies showed that EBS6/AtOS9, a well-established ERAD component, relies on its interaction with the ER membrane-anchored EBS5/AtHrd3 for its ER localization (Su et al. 2012). Similarly, SDF2 (stromal cell-derived factor-2) also forms a protein complex with ERdj3B and BIP for its ER retention (Nekrasov et al. 2009). Identification of such TIN1-interacting proteins could help us understand the likely biochemical function of TIN1 in coping with increased accumulation of misfolded proteins in the ER.

Our initial identification of TIN1 as an Hrd1-associated protein suggested a potential role of TIN1 in ERAD; however, the results presented in this study indicate that TIN1 unlikely participates in the ERAD-mediated degradation of the 2 ER-retained mutant receptors, bri1-5 and bri1-9. Nevertheless, we cannot exclude the possibility that TIN1 takes part in the degradation of other, yet unidentified physiological substrates, particularly in pollen, where TIN1 accumulates to high levels and its loss results in thermosensitive developmental phenotypes under elevated temperatures. It is worth noting that the thermosensitive phenotype of the *tin1* mutant was considerably weaker than that observed in Arabidopsis mutants defective in SEC62 or simultaneously lacking calnexin1 (CNX) and 3 calreticulins (CRTs), producing collapsed pollen grains under normal growth conditions (Vu et al. 2017; Hu et al. 2020; Mitterreiter et al. 2020). Sec62 was recently characterized as an ER-phagy receptor (Hu et al. 2020) and could function in a plant Sec61 translocon as the only Arabidopsis homolog of the yeast and mammalian Sec62 (Mitterreiter et al. 2020), which is required for importing newly synthesized proteins into the ER (Gemmer and Förster 2020). CNXs and CRTs are important lectin-type chaperones critical for protein folding (Caramelo and Parodi 2015). By contrast, its mild heat-sensitive male sterility resembles that of Arabidopsis mutants defective in AtERdj3A or ERdj3B (Yang et al. 2009; Yamamoto et al. 2020), both of which function as BIP-cochaperones promoting protein folding (Nekrasov et al. 2009; Ma et al. 2015). Therefore, we suspected that TIN1 likely functions as an accessory factor of the ER proteostasis system, helping plants manage the ER stress triggered by chemicals in vegetative tissues and increased secretory demands during pollen development, especially under elevated temperatures.

**Figure 8.**
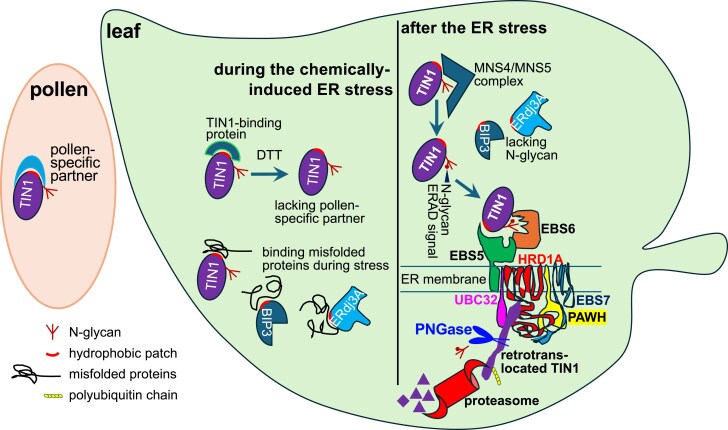
A simple model for the differential stability of 3 UPR proteins. During pollen development, TIN1 interacts with certain pollen-specific binding partners to adopt its native conformation. Upon chemically induced ER stress, TIN1 accumulates in vegetative tissues where it may become “misfolded” in the absence of its pollen-specific partners. ER-stress-inducing chemical may also disrupt TIN1's interaction with its partner(s), exposing surface hydrophobic residues. Alternatively, TIN1 may directly associate with misfolded proteins or act in concert with ER-resident chaperones such as BIP3 and ERdj3A, thereby contributing to ER proteostasis regulation. Upon removal of the stress-inducing chemicals, “misfolded” TIN1 with surface-exposed hydrophobic patches is recognized by the folding-sensitive MNS4/MNS5 mannosidase complexes, which generate a conserved N-glycan-based ERAD signal. This N-glycan signal is detected by EBS6/AtOs9, while the hydrophobic patch is recognized by EBS5/AtHrd3. Together, they coordinate the recruitment of TIN1 to the Hrd1-containing ERAD machinery, which includes UBC32, EBS7, and PAWHs. The HRD1 complex mediates retrotranslocation and ubiquitination of unfolded TIN1, targeting it for proteasome degradation in the cytosol—a process facilitated by PNGase-mediated deglycosylation. In contrast, ER chaperones such as BIP3 and ERdj3A lack N-glycosylation and therefore do not generate an N-glycan ERAD signal. This may explain their relative stability during the recovery phase following the chemically induced ER stress.

Our study led to the conclusion that TIN1 is an endogenous ERAD substrate that relies on its N-glycans and the Arabidopsis Hrd1-containing ERAD complex for its post-ER-stress degradation (Fig. 8). Our experiments also suggested that TIN1 is rapidly degraded after its translation from heat stress-induced *TIN1* transcripts. We found that the heat treatment resulted in >40-fold induction of the *TIN1* transcript in vegetative tissues (Supplementary Fig. S1E), yet the TIN1 protein was below the detectable threshold by our anti-TIN1 antibody (Supplementary Fig. S17A). By contrast, a similar heat treatment with the *hrd1a hrd1b* mutant seedlings or MG132-treated wild-type seedlings caused an easily observable accumulation of the TIN1 protein (Supplementary Fig. S17A and B). Previous studies suggested 2 Arabidopsis proteins as potential endogenous ERAD substrates, AtOS9/EBS6 and UBC32 (Cui et al. 2012; Su et al. 2012; Chen et al. 2016, 2017). Both proteins are involved in ERAD, with AtOS9/EBS6 functioning as a substrate recruitment factor that specifically recognizes a uniquely-demannosylated N-glycan on a terminally-misfolded glycoprotein (Huttner et al. 2012; Su et al. 2012), while UBC32 being an ER membrane-anchored E2 ubiquitin that works together with the ER membrane-anchored E3 ligases (Cui et al. 2012). The discoveries of EBS6/AtOS9 and UBC32 being the endogenous ERAD substrates support the idea of “ER tuning”, a term that was coined to describe the biochemical mechanism that regulates the abundance of the ERAD components to cope with dynamic changes of misfolded proteins in the ER in response to developmental cues and environmental signals. Select ERAD regulators are highly unstable in unstressed cells and during the stress recovery phase (Bernasconi and Molinari 2011). Our study revealed that ERAD is also used to degrade UPR-induced glycoproteins upon removal of ER stress; however, further studies are needed to determine whether or not rapid degradation upon removal of ER stress is a unique property of TIN1 or applies to other known UPR-induced proteins. In mammalian cells, ERAD is known to regulate the abundance of 2 key sensors of the mammalian UPR mechanism, IRE1α and ATF6 (Horimoto et al. 2013; Sun et al. 2015), but little is known about whether ERAD is also utilized to return the abundance of UPR-induced proteins to their pre-stress levels. Recent studies, however, suggested that a unique form of autophagy, known as “recov-ER-phagy” is the most likely mechanism to not only reduce the abundance of UPR-induced ER proteins but also decrease the size of the ER that is expanded during the ER stress to increase ER folding capacity (Loi and Molinari 2020). It should be recognized that a similar SEC62-mediated “recov-ER-phagy” mechanism also operates in plant cells to facilitate ER-stress recovery and that loss-of-function *sec62* mutations in Arabidopsis result in severe pollen phenotypes at normal temperature (Hu et al. 2020; Mitterreiter et al. 2020). Identification of additional endogenous ERAD substrates will certainly shed more light on the physiological roles of the conserved degradation mechanisms in plant growth and development.

What signal directs the stress-induced TIN1 protein into the ERAD pathway? Our study indicates that TIN1 degradation depends on a conserved N-glycan signal, as treatment with Kif (a specific alpha1,2-mannosidase inhibitor) or genetic elimination of the 2 ER-localized alpha1,2-mannosidases MNS4 and MNS5 effectively inhibited TIN1 degradation. We further demonstrated that TIN1 degradation requires both EBS6/AtOs9 and EBS5/AtHrd3, the Arabidopsis homologs of the Yos9/OS9 and Hrd3/Sel1L, respectively. In yeast and mammalian cells, Yos9/OS9 and Hrd3/Sel1L act together as a dual recognition module: Yos9/OS9 binds the conserved N-glycan signal, while Hrd3/Sel1L detects surface-exposed hydrophobic patches, thereby enhancing the selectivity and efficacy of ERAD to eliminate only irreparable misfolded glycoproteins (Fig. 8). Recent studies in yeast and mammalian cells have shown that Htm1/EDEM, orthologs of MNS4/MNS5, form a disulfide-linked complex with a protein disulfide isomerase (PDI) to catalyze the C-branch alpha1,2-mannose trimming of misfolded glycoproteins, with PDI serving as a protein-folding sensor for exposed hydrophobic residues. Therefore, we hypothesize that a surface-exposed hydrophobic patch is the most likely degron that commits TIN1 to the ERAD pathway. Consistent with our hypothesis, the AlphaFold-predicted TIN1 structure contains a central β-saddle of 2 β-sheets sharing a long curved β-strand, a lateral open-ended β-barrel with a hydrophobic cavity, and 5 flexible regions carrying hydrophobic residues, which may be involved in conformation flexibility, protein interactions, or ERAD targeting (Supplementary Fig. S20).

Although DTT treatment is unlikely to alter the structure of the 424-amino acid TIN1 polypeptide lacking any Cys residue (Supplementary Fig. S5), it does trigger the misfolding of numerous proteins, including some TIN1-client proteins. Upon removal of the ER-stress-inducing chemical, many of these misfolded client proteins are eliminated, most likely through chaperone-assisted refolding or ERAD-mediated degradation. The disappearance of those TIN1-clients likely exposes hydrophobic residues of TIN1, which may then be recognized by yet to be defined folding-sensitive MNS4/5 complexes to generate the conserved N-glycan ERAD signal. Such an N-glycan signal and the exposed hydrophobicity are subsequently recognized by EBS6/AtOs9 and EBS5/AtHrd3, respectively, ultimately targeting TIN1 for ERAD. Alternatively, DTT treatment may simply disrupt the interaction between TIN1 and its binding partners that normally retain TIN1 in the ER or cooperate in regulating ER proteostasis, thereby exposing hydrophobic residues. Another possibility is that TIN1 normally binds to certain pollen-specific non-UPR proteins to support the higher secretory demand during pollen development. The absence of these pollen-specific binding partners in vegetative tissues may cause TIN1 to adopt a “misfolded” conformation with a surface-exposed hydrophobic patch that could be detected by the ERAD machinery for its rapid degradation. Consistently, TIN1-GFP expressed in *p35S::TIN1-GFP* transgenic Arabidopsis lines was found to be unstable, which could be caused by an insufficient amount of its TIN1-binding partners to balance stoichiometrically excessive TIN1-GFP or the absence of the hypothesized pollen-specific interactors. By contrast, its homolog TIL1, which carries 2 Cys residues and has a similar AlphaFold-predicted structure (Supplementary Fig. S20), is a constitutively expressed protein that could have sufficient amounts of its binding partners in the vegetative tissues to shield its hydrophobic regions. It is worth mentioning that EBS6/AtOS9 becomes unstable and undergoes ERAD-mediated degradation when its binding partner EBS5/AtHrd3A is mutated or is insufficiently relative to transgenically overproduced EBS6/AtOS9 (Su et al. 2012; Chen et al. 2017), while the endogenous EBS6/AtOS9 is highly stable and remains detectable even 24 h after CHX treatment in wild-type Arabidopsis seedlings (Supplementary Fig. S21) (Su et al. 2012). Therefore, identification of TIN1-binding partners, particularly in pollen, should be one of the priorities for future TIN1 studies.

Unlike TIN1, BIP3 and ERdj3A contain 4 and 5 Cys residues, respectively (Supplementary Fig. S22, A to D), and would therefore be expected to be more susceptible to DTT-induced protein misfolding. These 2 proteins were previously thought to work as the chaperone/co-chaperone pair to refold or degrade misfolded proteins (Ma et al. 2015). Surprisingly, however, both proteins remained stable after the DTT/CHX treatment (Fig. 7). This revelation seems inconsistent with our model that exposed hydrophobic residues are the primary signal for the degradation of the UPR-induced TIN1 but not UPR-induced BIP3 or ERdj3A/TMS1 in DTT-treated Arabidopsis seedlings. The differential stability of these 3 UPR-induced proteins during the stress recovery phase could be attributed to N-glycosylation, which was shown to be important for the TIN1 degradation (Fig. 4). Indeed, N-glycosylation prediction using NetNGlyc-1.0 (https://services.healthtech.dtu.dk/services/NetNGlyc–1.0/) suggested 0 and 1 glycosylation sites in BIP3 and ERdj3A, respectively, while Endo H analysis of the 2 DTT-induced UPR proteins confirmed that neither protein is glycosylated (Supplementary Fig. S22E). The Endo H results were consistent with the same mobility of BIP3 and ERdj3A bands between seedlings treated with DTT and seedlings treated with the N-glycosylation inhibitor TM (Fig. 7), which induced the accumulation of glycosylated and nonglycosylated forms of TIN1 with differing mobility (Supplementary Fig. S2A). Analysis of protein stability of additional UPR-induced Cys-containing glycoproteins will help clarify whether both N-glycosylation and surface-exposed hydrophobicity are required for degrading a UPR-induced protein during the stress recovery phase. It will also be important to test whether treatment with DTT or TM, which had no effect on the *TIL1* transcript abundance, could make TIL1 an ERAD substrate, given that this TIN1 homolog has 2 Cys residues and 7 predicted N-glycosylation sites (Supplementary Fig. S20). Finally, quantitative proteomic analyses will be valuable for identifying additional UPR-proteins that are rapidly degraded via the Hrd1 ERAD pathway during the stress recovery phase.

## Materials and methods

### Plant materials and growth conditions

Most of the Arabidopsis (*A. thaliana*) wild-type, mutants, and transgenic lines are in the Columbia (Col-0) ecotype, except for mutants and transgenic lines carrying the *bri1-5* mutation that was in the Wassilewskija-2 (Ws-2) ecotype. The Arabidopsis mutants used in this study include, *bri1-9* (Jin et al. 2007), *ebs5* (Su et al. 2011), *ebs6* (Su et al. 2012), *ebs7-1* (Liu et al. 2015)*, hrd1a hrd1b* (Su et al. 2011), *pawh1 pawh2* (Lin, et al. 2019), *ire1a-2*, *ire1b-4* (Nagashima et al. 2011), and *s2p*, *bzip28*, *bzip60* (Moreno et al. 2012). The T-DNA insertional mutants *CS411789* (*tin1-1*) and *CS808001* (*til1-1*) were obtained from the Arabidopsis Biological Resource Center (ABRC) at Ohio State University. Seeds were surface sterilized using the ethanol-washing protocol, and germinated seedlings were grown at 22 °C in a growth chamber or a growth room under long-day (16h-light/8h-dark) photoperiodic conditions.

### Generation of transgene constructs and transgenic plants

A 2-kb genomic DNA fragment upstream from the start codon of *TIN1* as the *TIN1* promoter was amplified from genomic DNAs of the wild-type Arabidopsis Col-0 seedlings using the *pTIN1-GUS* primer set (Supplementary Table S1) and cloned into *pCambia1300* (https://www.snapgene.com/plasmids/plant_vectors/pCAMBIA1300) to generate *pCambia1300-proTIN1::GUS* transgenes. The *35S::TIN1-GFP* and *35S::TIL1-GFP* transgene was created by cloning a 1,272-bp *TIN1* and 1,185-bp *TIL1* cDNA fragment amplified from the first-strand cDNAs converted from total RNAs of the wild-type Arabidopsis seedlings using the *cTIN1-GFP* and *cTIL1-GFP* primer sets (Supplementary Table S1) into the *pCambia1300p35S::C-GFP* vector, respectively. To generate the *pTIN1::TIN1-GFP*, a 3.3-kb *TIN1* genomic fragment containing the *TIN1* promoter was PCR amplified from the genomic DNAs of the wild-type Arabidopsis (Col-0) seedlings using the *gTIN1-GFP* primer set (Supplementary Table S1) and cloned into the *pCambia1300C-GFP* vector. The different N-glycan-mutant transgenes of *TIN1* were amplified from the *pTIN1::TIN1-GFP* plasmid using different site-directed mutagenesis primer sets (Supplementary Table S1). All created transgenes were fully sequenced to ensure no PCR-introduced sequence error and were individually transformed into the *Agrobacterium tumefaciens* strain *GV3101* by electroporation, and the resulting *Agrobacterial* strains were subsequently used to transform into Arabidopsis plants using the floral-dipping method (Clough and Bent 1998).

### Expression of fusion proteins and generation of antibodies

The first-strand cDNA preparation derived from total RNAs of wild-type Arabidopsis seedlings and the *TIN1-antigen* primer sets (Supplementary Table S1) were used to amplify a 1,191-bp *TIN1* cDNA fragment encoding a 396-AA (28-424) polypeptide. The amplified cDNA fragment was cloned into *pET-28a* (Novagen) and *pGEX-4T-1* (GE Healthcare) vectors, which were subsequently transformed into BL21-competent cells. The induction of His- and GST- fusion proteins and their subsequent purification using TALON Metal Affinity Resin (Clontech Laboratories) and Glutathione Sepharose 4 Fast Flow beads (GE Healthcare), respectively, were carried out following the manufacturers' recommended protocols. The purified His-TIN1 fusion protein was used to generate a custom anti-TIN1 antibody at MBL Beijing Biotech Co. (http://www.mbl-chinawide.cn), while the purified GST-TIN1 fusion protein was used to affinity-purify the custom-made anti-TIN1 antibody using an online protocol with nitrocellulose membrane (http://post.queensu.ca/∼chinsang/lab-protocols/antibody-purification.html). The specificity of the purified anti-TIN1 antibodies was analyzed by immunoblotting with total proteins extracted from DTT-treated 10-d-old seedlings of wild-type Arabidopsis plants and *tin1* mutants (Supplementary Fig. S2B). The antibodies to BIP3 and ERdj3A/TMS1 were custom-generated and affinity-purified against the peptides, C^649^DPVIKSVYEKTEGENED^666^ and L^556^NGDIQFTKTRQKPQIK^572^, respectively, at ABclonal (www.abclonal.com.cn).

### Histochemical GUS staining assay

For histochemical GUS staining, 10-d-old *proTIN1::GUS* transgenic Arabidopsis seedlings were immersed into the GUS staining solution (0.1 m sodium phosphate buffer, 10 mm EDTA [pH 8.0], 0.5 mm K_3_Fe(CN)_6_, 0.5 mm K_4_Fe(CN)_6_, 1 mm X-glucuronide, 0.1% Triton X-100), vacuumed for 15 min, and subsequently incubated at 22 °C with or without ER-stress chemicals (5 *μ*g/mL TM, 2 mm DTT, or 5 mm AZC) for overnight. GUS-stained tissues were dehydrated with 75% ethanol and observed by a ZEISS Stereo Discovery V8 microscope.

### Sucrose density-gradient centrifugation

Sixteen grams of 10-d-old Arabidopsis seedlings were ground in liquid N_2_ into a fine powder and immediately extracted by the homogenization buffer (50 mm Tris–HCl [pH 8.2], 20% [v/v] glycerol, 1 mm phenylmethylsulphonyl fluoride [PMSF, Sigma], 2 mm ethylenediaminetetraacetic acid [EDTA], 1 mm DTT, 2 protease inhibitor cocktail tablets (Roche) per 100 mL solution) at 4 °C. The protein extracts were first filtered through Miracloth (CalBiochem) to remove insoluble plant debris and subsequently centrifuged at 5,000 × *g* for 5 min at 4 °C to remove cellular debris and organelles. The supernatant was further centrifuged at 100,000 × *g* for 45 min to pellet the microsomes, which were resuspended in 1 mL resuspension buffer (25 mm Tris–HCl [pH 7.5], 10% [w/v] sucrose, 1 mm PMSF, 2 mm EDTA, 1 mm DTT, 2 protease inhibitor cocktail tablets [Roche] per 100 mL). The microsomal resuspension was loaded onto the top of a 11 mL 20% to 50% (w/w) sucrose gradient in 10 mm Tris–HCl (pH 7.5), 2 mm EDTA, 1 mm DTT, 0.1 mm PMSF, and centrifuged at 100,000 × *g* for 16 h at 4 °C. After centrifugation, 14 fractions (0.8 mL/each) were manually collected, and 50 *µ*L protein sample for each fraction was mixed with 2× SDS buffer, 37 °C warming for 1 h, separated by 10% SDS/PAGE, and analyzed by immunoblotting. For the Mg^2+^-plus experiments, 5 mm MgCl_2_ was added to the buffers of homogenization, resuspension, and ultracentrifugation. To determine if TIN1 is an ER luminal protein, the microsomal pellets of the wild-type seedlings were resuspended in solutions of 0.1 m NaCl, 0.1 m Na_2_CO_3_, 1% (v/v) Triton X-100 (Sigma) or 1% (v/v) Nonidet P-40 (Roche) and incubated at 4 °C for 4 h. The resuspended microsomes were centrifuged at 100,000 × *g* at 4 °C for 60 min to collect the supernatants (soluble fraction) and the pellets (P), which were mixed with 2× SDS buffer, boiled for 10 min, separated by 12% SDS-PAGE, and analyzed by immunoblotting with antibodies against TIN1, BIP, or EBS7.

### Chemical treatment of Arabidopsis seedlings and subsequent immunoblot analyses

To analyze ER-stress induction, 10-d-old Arabidopsis seedlings were carefully transferred into liquid 1/2 mS medium supplemented with 5 *µ*g/mL TM (BioMol), 2 mm DTT (Sigma), and 5 mm AZC (TCI Shanghai), incubated for varying durations, and subsequently harvested into liquid nitrogen for immediate protein extraction or stored in a −80 °C freezer. To study protein stability, 10-d-old Arabidopsis seedlings were pretreated with 2 mm DTT in liquid 1/2 mS medium for 4 h and then carefully transferred into liquid 1/2 mS medium containing 180 *µ*M CHX (Sigma) with or without 2 mm DTT, incubated for varying durations, and harvested into liquid nitrogen for protein extraction. To study the impact of Kif and MG132 treatment on protein stability, 10-d-old Arabidopsis seedlings were treated with 50 *µ*M Kif (Abcam) or 80 *µ*M MG132 (Sigma) in liquid 1/2 MS medium for varying durations and then harvested into liquid nitrogen for protein extraction. To study the impact of Endo H treatment on the electromobility of a given protein, 30 mg of 10-d-old Arabidopsis seedlings grown on 1/2 MS-agar medium were harvested into liquid nitrogen and ground thoroughly in 150 *μ*L 2× SDS loading buffer. An aliquot of 44 *μ*L protein extracts was mixed with 1 *μ*L Endo H-Hf and 5 *μ*L 10× G5 buffer (New England Biolab), incubated at 37 °C for 1 h, and subsequently analyzed by SDS-PAGE and immunoblotting.

### Expression and confocal analysis of TIN1/TIL1 fusion proteins

Transgenes of *p35S::TIN1-GFP* and *p35S::TIL-GFP* were generated (see above) for analyzing the TIN1/TIL1 subcellular localization patterns. The plasmid *p35S::RFP-HDEL* was the same as previously described (Liu et al. 2015). These plasmids and the corresponding empty vectors, after verifying no PCR-introduced error by sequencing, were individually transformed into the *A. tumefaciens* strain *GV3101*. The *p35S::RFP-HDEL*-carrying *GV3101* cells were mixed with the *p35S::TIN1-GFP* or *p35S::TIL1-GFP*-transformed *GV3101* strain and co-transformed into leaves of 3-wk-old tobacco (*N. benthamiana*) plants by the agro-infiltration method (Yang et al. 2000). Two days after infiltration, the transformed tobacco leaves were examined by confocal microscopy on a Leica SP8 (with LAS AF software, Leica Microsystems) for the subcellular localization patterns of GFP-tagged TIN1/TIL1 and the ER-localized RFP-HDEL. GFP and RFP were excited by using 488- and 542-nm laser light, respectively.

### RNA analyses

Ten-day-old Arabidopsis seedlings grown on 1/2 mS agar medium supplemented with or without certain chemicals were harvested and ground in liquid nitrogen into a fine powder, and their total RNAs were extracted using the RNeasy Plant Mini Kit (QIAGEN). One microgram of the purified total RNAs was treated with RNase-free DNase I (TIANGEN) and subsequently reverse transcribed into the first-strand cDNAs by the iScript cDNA synthesis Kit (Bio-Rad). The resulting cDNA preparations were used for traditional PCR or RT-qPCR analysis with gene-specific oligonucleotides listed in Supplementary Table S1. The qPCR assays were performed on the CFX96 Real-Time System (Bio-Rad) with SYBR GREEN PCR Master Mix (Bio-Rad) following the manufacturer's instructions. Three biological replicates with 3 technical repeats were conducted for each target mRNA, with the *ACTIN8* cDNA used as an internal reference to calculate the relative transcript abundance.

### Scanning electron microscopic analysis of the pollen morphology

Arabidopsis pollen grains were mounted on stubs, coated with palladium-gold in a sputter coater (Emitech K575X, England), and then examined using a scanning electron microscope (Hitachi_S4700_FESEM, Japan) with an acceleration voltage of 5 kV.

### Accession numbers

Sequence data from this article can be found in the GenBank/EMBL data libraries under accession numbers: TIN1, NP_201256; TIL1, NP_564503; BRI1, NP_195650; EBS6, Q8GWH3.1; EBS7, NP_567837; BIP3, Q8H1B3.1; and ERdj3A, Q9SR96.1.

## Supplementary Material

kiaf489_Supplementary_Data

## Data Availability

The data that support the findings of this study are available from J.L. (li-jianming@hkbu.edu.hk) upon reasonable request.

## Supplementary data

The following materials are available in the online version of this article.

**Supplementary Figure S1.** TIN1 is induced by ER stress and other stresses.

**Supplementary Figure S2.** The specificity test of a custom-made anti-TIN1 antibody.

**Supplementary Figure S3.** TIN1 is an ER luminal protein.

**Supplementary Figure S4.** TIN1 and TIL1 play no significant role in ERAD of bri1-5 and bri1-9.

**Supplementary Figure S5.** The presence of the TIN1–TIL1 pair in green organisms.

**Supplementary Figure S6.** TIL1 likely contains a signal peptide.

**Supplementary Figure S7.** TIL1 is also an ER-localized protein that does not respond to ER stress.

**Supplementary Figure S8.** Identification of a T-DNA insertional *til1-t* mutant.

**Supplementary Figure S9.** Overexpression of *TIN1* or *TIL1* had little impact on the *bri1-9* mutant.

**Supplementary Figure S10.** Neither *tin1-1* nor *TIN1*-overexpression had any observable effect on the pollen grain at 22 °C.

**Supplementary Figure S11.**  *tin1-1* exhibits the defective pollen surface phenotype at 30 °C.

**Supplementary Figure S12.** Gradual reduction of the transcript abundance of 3 UPR genes following DTT removal.

**Supplementary Figure S13.** The AZC-induced TIN1 protein is also rapidly degraded.

**Supplementary Figure S14.** The TIL1-GFP fusion protein is very stable.

**Supplementary Figure S15.** Conservation of the 3 glycosylated Asn residues between TIN1 and its homologs in green organisms.

**Supplementary Figure S16.** The effect of the known ERAD mutations on the *TIN1* transcript and TIN1 protein.

**Supplementary Figure S17.** The impact of heat stress, MG132, and *hrd1a hrd1b* double mutation on the TIN1 stability.

**Supplementary Figure S18.** The degradation of the AZC-induced TIN1 also involves the EBS7-PAWH1/2-Hrd1 machinery.

**Supplementary Figure S19.**  *TIN1* is coexpressed with *BIP3* and *ERdj3A/TMS1*.

**Supplementary Figure S20.** The AlphaFold predicted structural models of TIN1 and TIL1.

**Supplementary Figure S21.** The endogenous EBS6/AtOS9 is a very stable protein.

**Supplementary Figure S22.** Both BIP3 and ERdj3A are nonglycosylated ER proteins.

**Supplementary Table S1.** Oligonucleotide primers used in the study.
