# Supplementary material for: The endoplasmic reticulum-associated degradation machinery selectively degrades stress-induced TIN1 during stress recovery
Source: Plant Physiol. 2025 Oct 6;199(2):kiaf489. doi: 10.1093/plphys/kiaf489 (PMC12548798; doi:10.1093/plphys/kiaf489)
Supplement: kiaf489_Supplementary_Data [file kiaf489_supplementary_data.pdf]

Supplementary Figures and Table

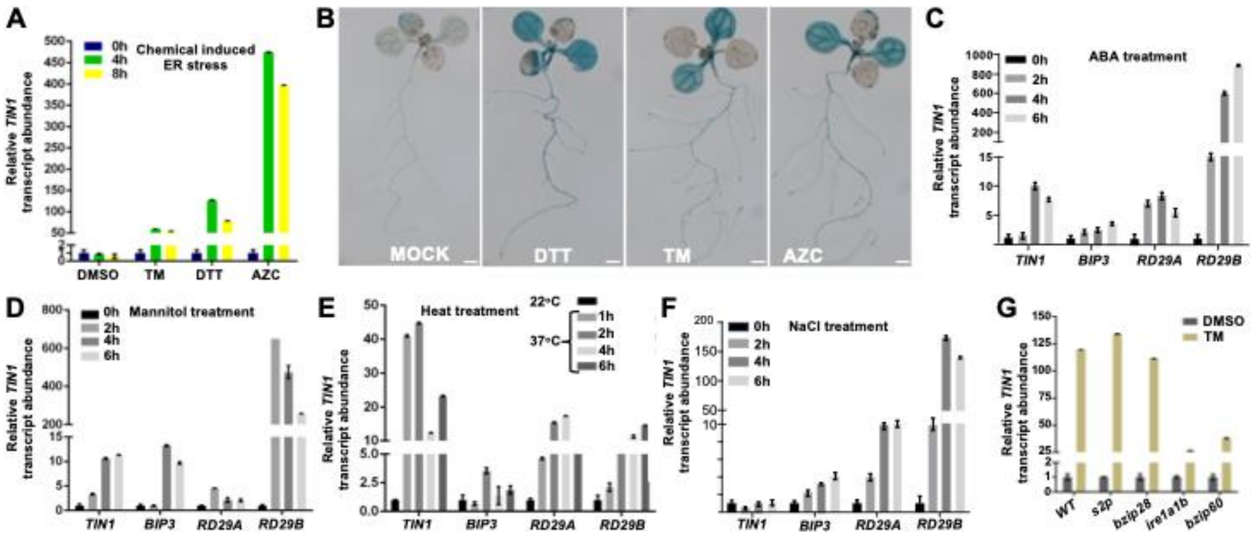

Supplementary Figure S1. TIN1 is induced by ER stress and other stresses.

A. Real-time reverse transcription-quantitative PCR (RT-qPCR) analysis of the *TIN1* transcript abundance in 10-day-old wild-type seedlings treated with or without 5 μg/mL tunicamycin (TM), 2 mM dithiothreitol (DTT), or 5 mM L-azetidine-2-carboxylic acid (AZC). Scale bars in all seedling images equal

B. Histochemical staining of 10-day-old mock/chemical-treated seedlings of a representative *pTIN1::GUS* transgenic line. The scale bar in each image equals 10 mm.

C-F. RT-qPCR analysis of the transcript abundance of *TIN1*, *BIP3*, *RD29A*, and *RD29B* genes. Both *RD29A* and *RD29B* genes were known to be induced by a variety of stresses (Yamaguchi-Shinozaki and Shinozaki, 1994) and were thus included as positive controls for our stress experiments.

G. RT-qPCR analysis of the *TIN1* transcript abundance in dimethyl sulfoxide (DMSO)/TM-treated 10-day-old seedlings of indicated genotypes. For **A** and **C-G**, the first-strand cDNAs derived from total RNAs isolated from 10-day-old light-grown wild-type Arabidopsis seedlings were used to perform the RT-qPCR analyses of the indicated genes with the oligonucleotide primers listed in **Supplementary Table S1**. Each bar is the relative value of the abundance of a cDNA target in treated Arabidopsis seedlings of various genotypes to that of the non/mock-treated seedlings. The value of each bar represents the average result

of three biological replicates each with three technical repeats. The *ACTIN8* cDNA was used as the internal reference. Error bars represent ±SD. In **A**, **C**-**G**, the average value for each analyzed gene with non-/DMSO-treated samples is set to 1.

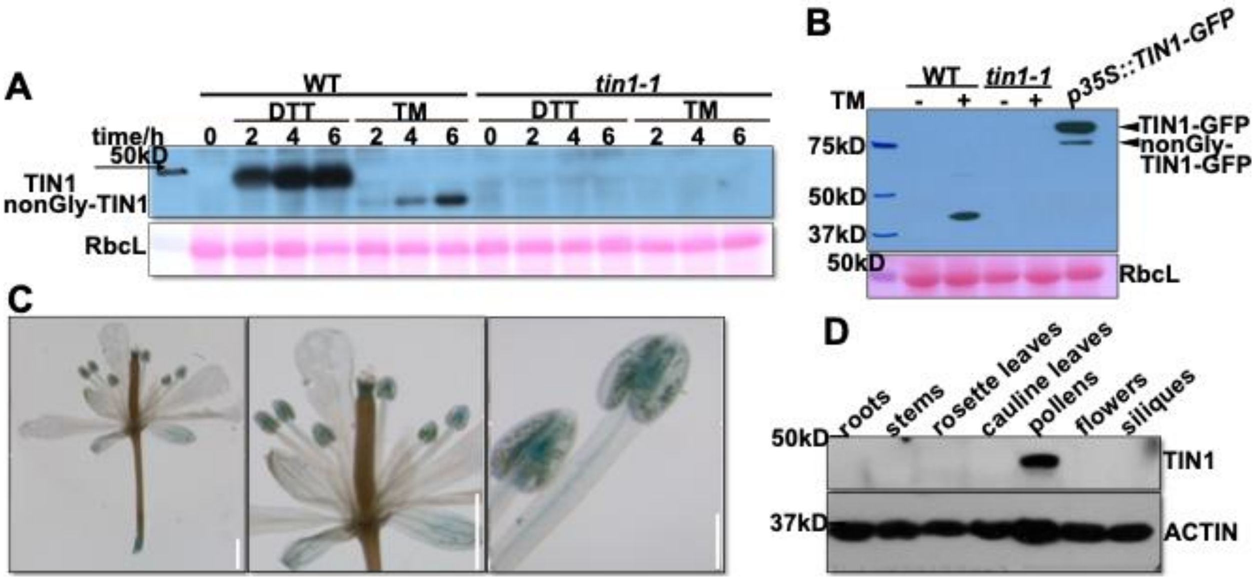

Supplementary Figure S2. The specificity test of a custom-made anti-TIN1 antibody.

A. Immunoblot analysis of the TIN1 protein in 10-week-old seedlings of the wild-type and *tin1-1* mutant treated with or without dithiothreitol (DTT) or tunicamycin (TM).

B. Immunoblot analysis of TIN1 protein in mock (dimethyl sulfoxide/DMSO)/TM-treated seedlings of the wild-type, *tin1-1*, and a representative *p35S::TIN1-GFP* transgenic line.

C. Histochemical staining of a developing flower of a representative *pTIN1::GUS* transgenic line. The images shown in the middle and on the right are enlarged crops of the image shown on the left. Scale bars for the three images are 1 mm, 1 mm, and 0.2 mm, respectively.

D. Immunoblot analysis of the TIN1 protein in different tissues of a mature soil-grown wild-type Arabidopsis plant.

In **A** and **B**, "nonGly" denotes the non-glycosylated form of the endogenous TIN1 or transgenically expressed TIN1-GFP fusion protein. The ponceau red-stained RbcL in **A** and **B** or immunoblotted ACTIN in **C** serves as the control for equal sample loading. The positions of molecular weight standards are shown on the left in each panel.

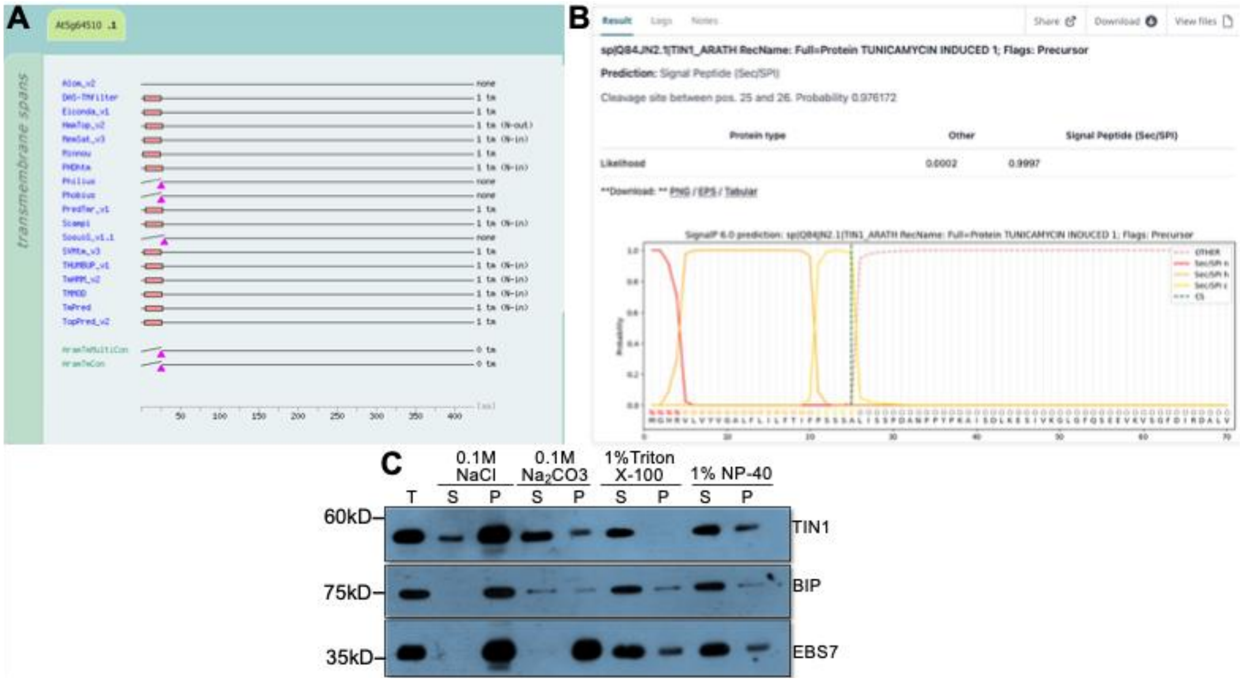

Supplementary Figure S3. TIN1 is an ER luminal protein

A. A screen shot of the topology prediction of TIN1 by ARAMEMNON (Schwacke and Flügge, 2018), a plant membrane protein database at https://aramemnon.botanik.uni-koeln.de.

B. The signal peptide prediction by signalP6.0 at https://aramemnon.botanik.uni-koeln.de/ (Teufel et al., 2022).

C. Immunoblot analysis of TIN1, BIP, and EBS7 of the soluble and pellet fractions following solubilization and re-centrifugation (at 100,000 *g*) of the microsomal pellets of the wild-type Arabidopsis seedlings using NaCl, Na_2CO_3, Triton X-100, and NP-40. T, the total microsomal proteins, S, the soluble fraction, and P, the pellet fraction. The positions of molecular mass standards are shown to the left of each blot image.

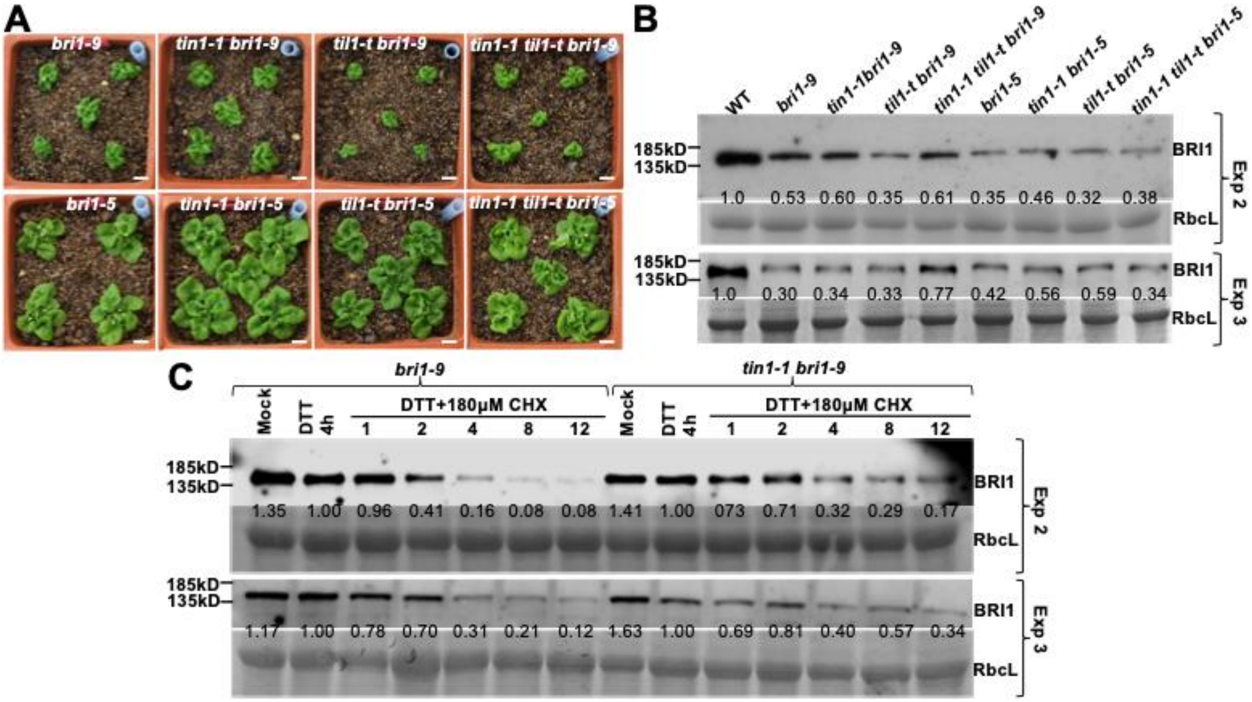

Supplementary Figure 4. TIN1 and TIL1 play no significant role in ERAD of bri1-5 and bri1-9.

A. Pictures of 2-week-old soil-grown plants of indicated genotypes. Scale bar in each image equals 10 mm. **B**. Two independent Immunoblot analyses of BRI1 abundance in 2-week-old seedlings of indicated genotypes**.** The numbers are the ImageJ-generated relative anti-BRI1 signals of various mutants to the wild-type after normalization with the RbcL signals. **C**. Two separate Immunoblot analyses of the bri1-9 stability of the *bri1-9* and *tin1-1 bri1-9* mutants. Two-week-old seedlings were pretreated with H_2O (mock) or 2 mM dithiothreitol (DTT) for 4 h and transferred into liquid ½ MS medium containing 2 mM DTT and 180 μM cycloheximide (CHX) for incubation. Equal amounts of seedlings were collected at indicated time points for extraction of total proteins, which were separated by SDS-PAGE and analyzed by immunoblotting with BRI1 antibody. The numbers represent the ImageJ-quantified relative anti-BRI1 signal intensity (normalized with the RbcL signals) in samples collected at the indicated time points relative to that of the wild-type seedlings, which was set to 1. In **B** and **C**, the ponceau red-stained RbcL serves as the loading control. The positions of molecular mass standards were indicated to the left of the blot images.

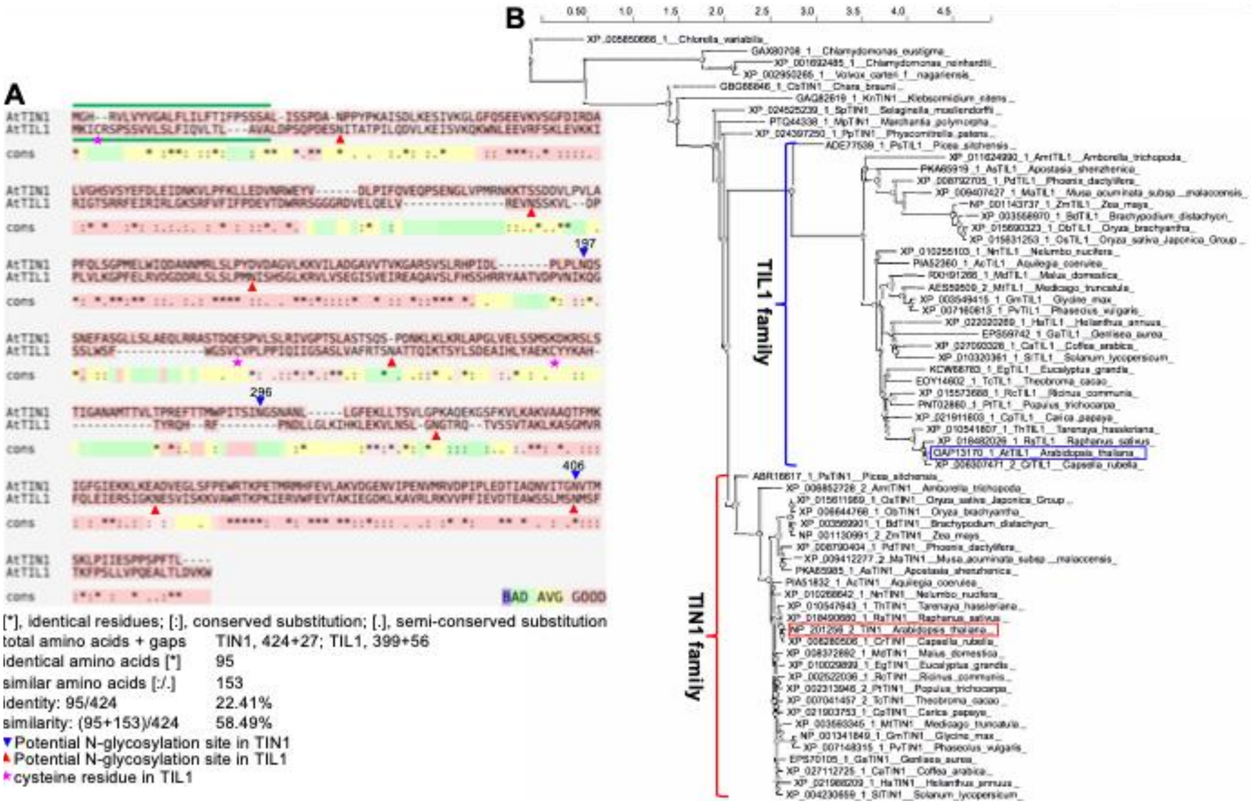

Supplementary Figure S5. The presence of the TIN1-TIL1 pair in green organisms.

A. Alignment of amino acid sequence between TIN1 and its Arabidopsis homolog TIL1. The color scale indicates the degree of amino acid conservation. The green bars indicated the predicted signal peptides of the two homologous proteins.

B. Phylogeny of the TIN1/TIL1 homologs in green organisms. Shown in the phylogeny tree are the protein accession numbers and the corresponding species names of the TIN1/TIL1 homologs. The Arabidopsis TIN1 and TIL1 are indicated by the red and blue box, respectively. The protein sequences were obtained from the GenBank and subsequently used for the phylogeny analysis at https://ngphylogeny.fr/ via the “One Click” mode (Lemoine et al., 2019). The scale on the top indicates the genetic distance (branch length) measured by the estimated number of nucleotide changes that have occurred along a given lineage over evolutionary time with a value of 1 on the scale representing 1 substitution per 100 nucleotides.

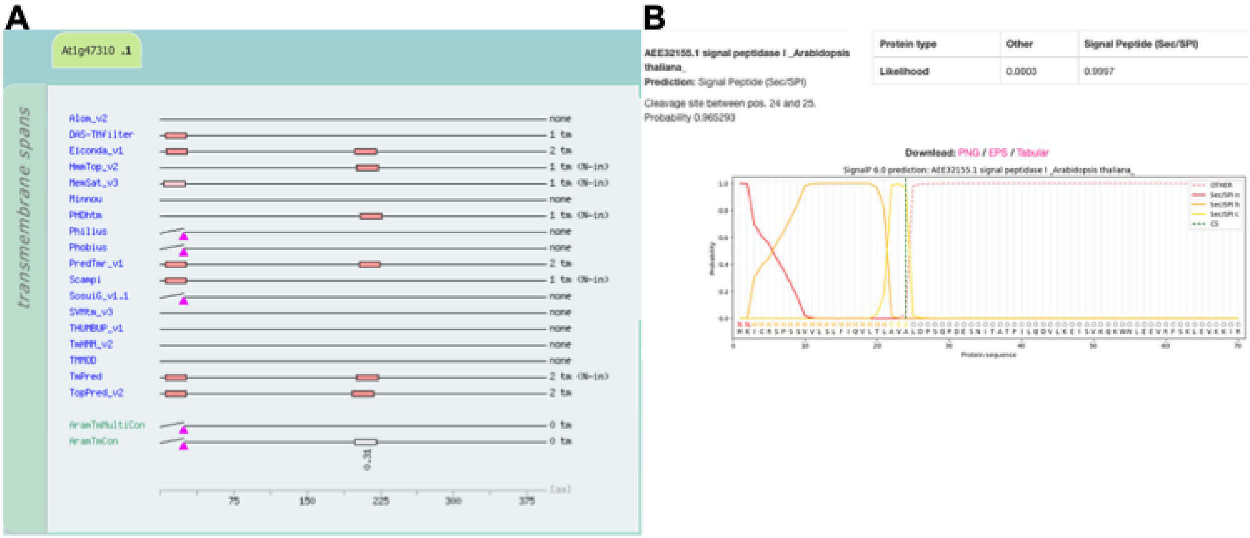

Supplementary Figure S6. TIL1 is also predicted to contain a signal peptide.

A. A screen shot of the topology prediction of TIL1 by ARAMEMNON (Schwacke and Flügge, 2018), a plant membrane protein database at https://aramemnon.botanik.uni-koeln.de.

B. The signal peptide prediction of TIL1 by signalP6.0 at https://aramemnon.botanik.uni-koeln.de/ (Teufel et al., 2022).

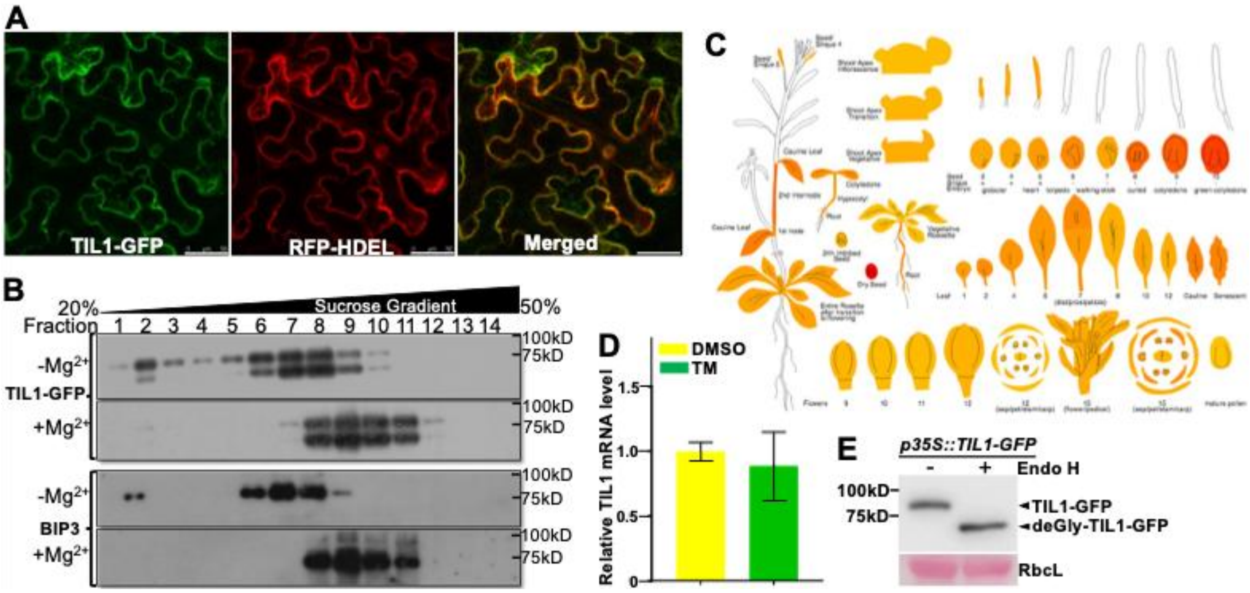

Supplementary Figure S7. TIL1 is also an ER-localized protein that does not respond to ER stress

A. Confocal analysis of the subcellular localization of a transiently expressed TIL1-GFP fusion protein in tobacco leaf epidermal cells. Shown here are green-fluorescent image of TIL1-GFP, red-fluorescent image of a widely used ER marker, RFP-HDEL, and a superimposed image of green and red fluorescent signals. Scale bar = 50 μm in each image.

B. A sucrose gradient ultracentrifugation analysis of the TIL1-GFP protein of a representative *p35S::TIL1-GFP* transgenic Arabidopsis line. Protein samples collected from a linear (20-50%) sucrose gradient in the absence or presence of Mg^{2+} were separated by SDS-PAGE and analyzed by immunoblotting with antibodies against GFP and BIP. The positions of molecular mass standards are shown to the right of the images.

C. The expression profile of *TIL1* in different tissues of growing Arabidopsis plants. This diagram was obtained from the ePlant website (http://bar.utoronto.ca/eplant/) (Fucile et al., 2011).

D. RT-qPCR analysis of the *TIL1* transcript in 2-week-old dimethyl sulfoxide (DMSO)/ tunicamycin (TM)-treated wild-type seedlings. The first-strand cDNAs derived from total RNAs isolated from 10-day-old DMSO/TM-treated Arabidopsis seedlings were used to perform the RT-qPCR analyses of the *TIL1* gene with the oligonucleotide primers listed in **Supplementary Table S1**. The bars show relative values of the *TIL1* transcript abundance in TM-treated seedlings to that of the DMSO-treated seedlings (set to 1) with *ACTIN8* used as an internal reference. The value of each bar represents the average result of three

biological replicates each with three technical repeats with the error bars indicating ±SD. **E**. Endoglycosidase H (Endo H) analysis of the GFP-tagged TIL1 protein. Total protein extracts of 2 week-old seedlings of a representative *p35S::TIL1-GFP* was subject to an Endo H assay followed by SDS-PAGE and immunoblot analysis with an anti-GFP antibody. The ponceau red-stained RbcL of a duplicated gel serves as the sample loading control. The numbers on the left are molecular mass standards and “deGly-” indicates deglycosylated form.

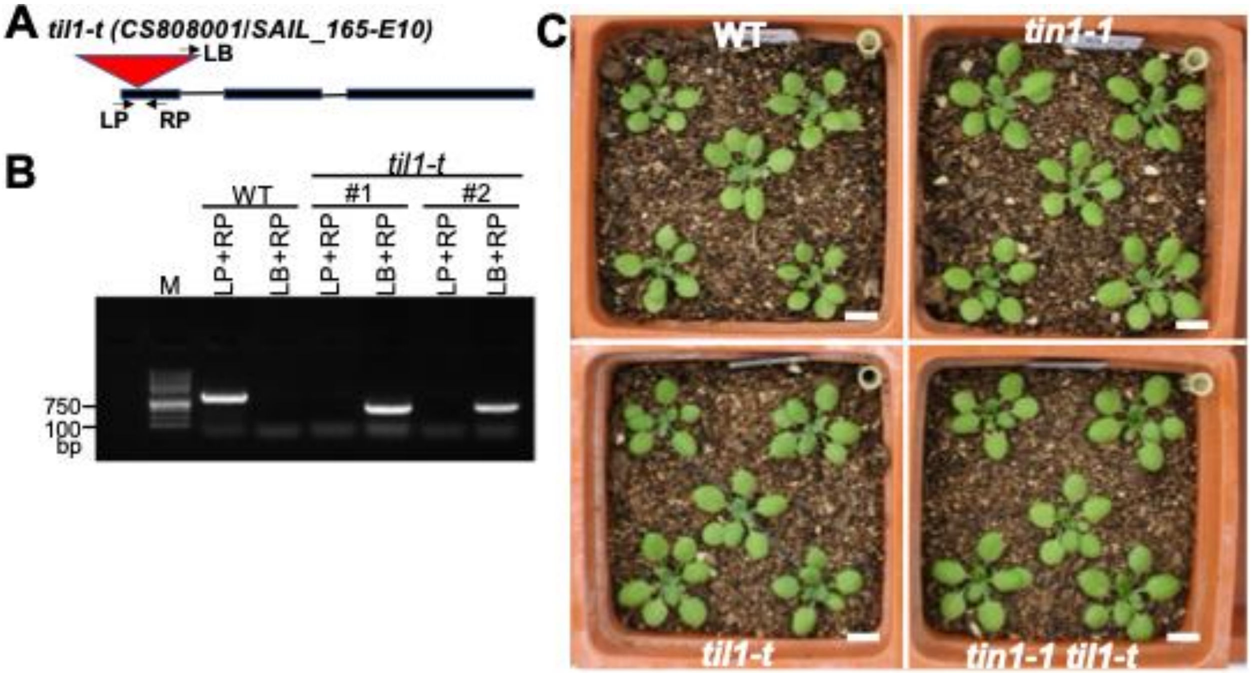

Supplementary Figure S8. Identification of a T-DNA insertional *til1-t* mutant.

A. Schematic presentation of the T-DNA insertion site in the 1st exon of the *TIL1* gene (*At1G47310*). The positions of the T-DNA insertion and the primers used for genotyping were indicated. The arrows indicate the positions of primers of left boarder of the T-DNA insertion (LB), left primer (LP), and right primer (RP) of the targeted DNA fragment.

B. PCR analysis of the T-DNA insertional mutation in the *til1-t* mutant. The positions of DNA length standards are shown on the left of the gel image. See **Supplementary Table S1** for oligonucleotide sequences of the three genotyping primers.

C. Pictures of 2-week-old soil-grown plants of wild-type, *tin1-1*, *til1-t*, and *tin1-1 til1-t*. Scale bar in each image equals 10 mm.

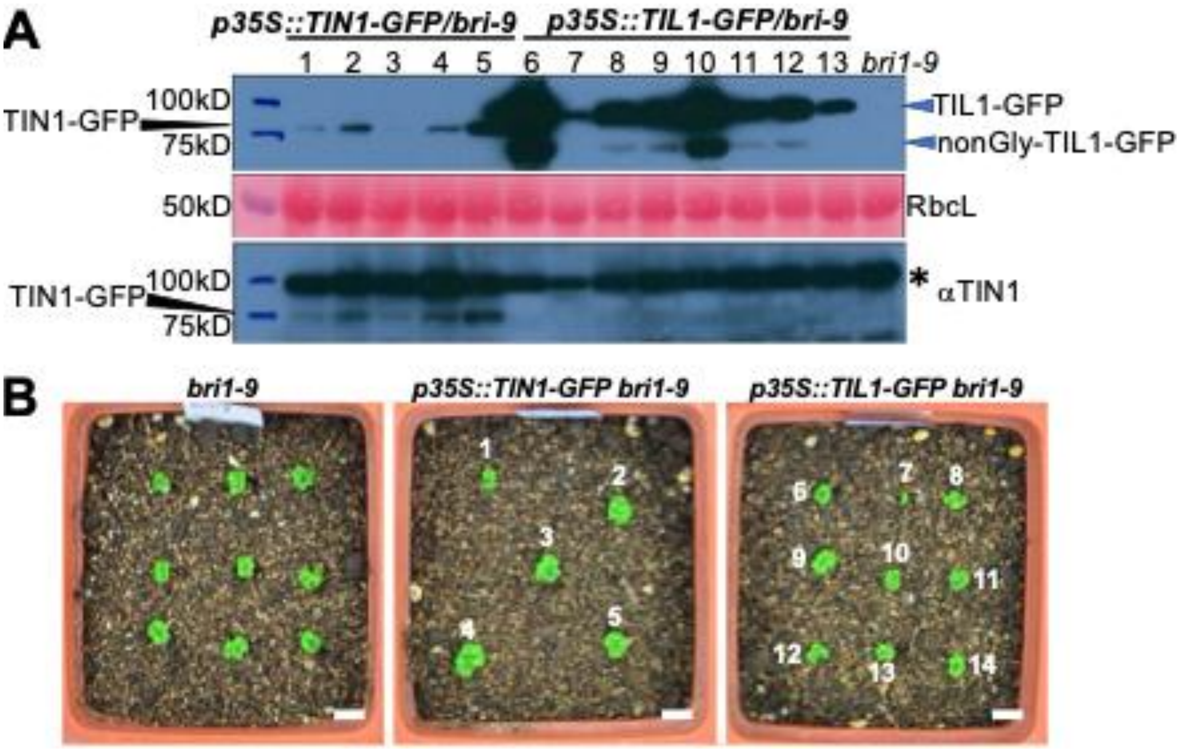

Supplementary Figure S9. Overexpression of *TIN1* or *TIL1* had little impact on *bri1-9* mutant.

A. Immunoblot analysis of the protein abundance of the *TIN1/TIL1-GFP* fusion proteins in independently-generated *p35::TIN1-GFP bri1-9* and *p35S::TIL1-GFP bri1-9* transgenic lines. The asterisk indicates the non-specific band recognized by the anti-TIN1 antibody while the anti-GFP antibody detected both glycosylated (TIL1-GFP) and non-glycosylated (nonGly-TIL1-GFP) forms of TIL1-GFP fusion protein. It should be noted that anti-TIN1 antibody did not cross react with the TIL1-GFP protein (see the lower immunoblot). The ponceau red-stained RbcL serves as the loading control for the above and below immunoblots, and the positions of molecular mass standards are shown to the left of blot/gel images. The star indicates a non-specific cross-reacting band in all tested samples.

B. Pictures of 2-week soil-grown plants of *bri1-9*, *p35S::TIN1-GFP bri1-9*, *p35S::TIL1-GFP bri1-9.* The numbers displayed in the middle and right images correspond to sample labels shown in **A**. Scale bar in each image equals 10 mm.

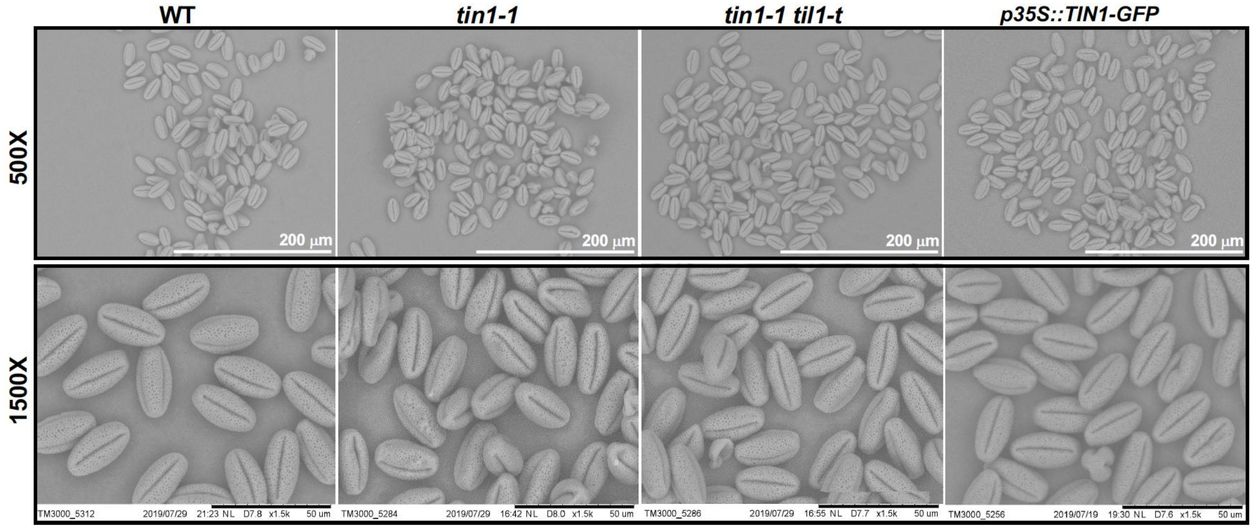

Supplementary Figure S10. Neither *tin1-1* nor *TIN1*-overexpression had any observable effect on the pollen grain.

Shown here are scanning electron micrographs (two different magnifications: the top panel, X500; the lower panel, X1500) of the pollen grains collected from the wild-type, *tin1-1*, *tin1-1 til1-t*, and a representative *p35S::TIN1-GFP* transgenic line. Scale bar in each image of the top row equals 200 μm while the black scale bar in each image of the bottom row equals 50 μm.

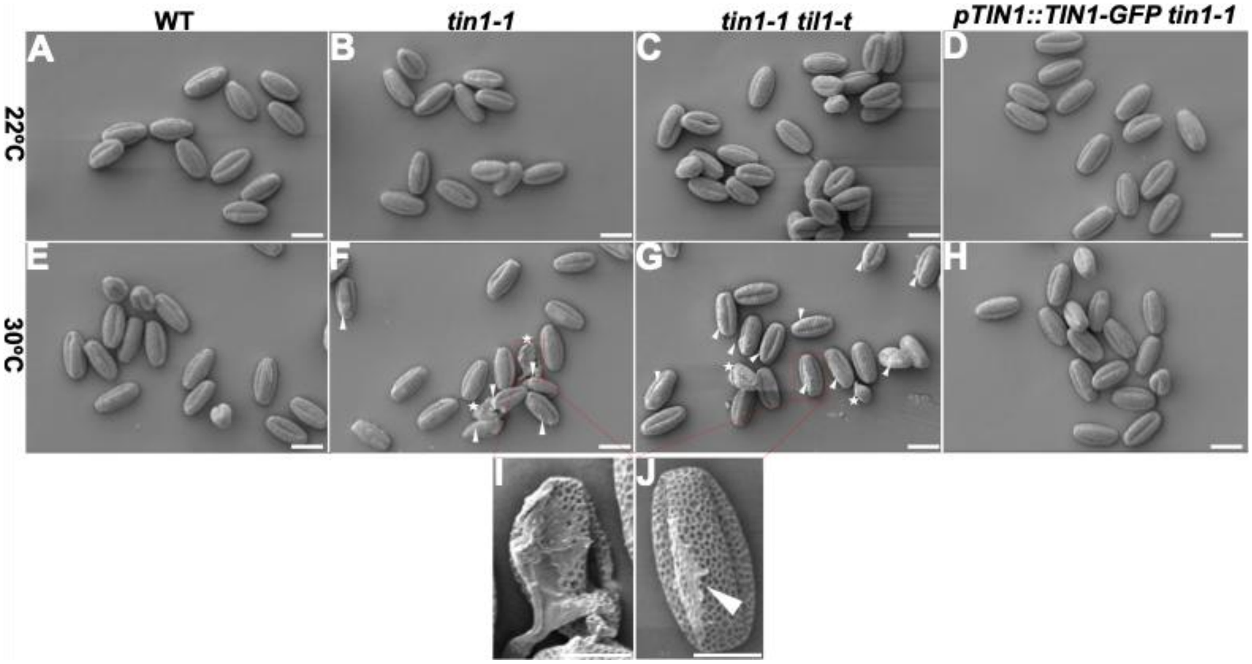

Supplementary Figure S11. The *tin1-1* exhibits the defective pollen surface phenotype at 30ºC.

Shown here are scanning electron micrographs of the pollen grains collected from the flowers formed entirely at 22ºC (**A-D**) or during the two-day 30ºC heat treatment (**E-J**) of the wild-type, *tin1-1*, *tin1-1 til1-t,* and a representative *pTIN1::TIN1-GFP* transgenic line. Seven-week-old 22ºC-grown flowering Arabidopsis plants with several formed siliques were transferred into a 30ºC growth chamber for 2-day growth and moved back to the 22ºC growth room. Pollens were collected from the newly opened anthers at the end of the 30ºC heat treatment and examined by scanning electron microcopy. Arrow heads mark white deposits on the pollen surface while stars label deformed/collapsed pollen grains. **I** and **J** show enlarged images of two individual pollen grains from panel **F** and **G**, respectively. Scale bar in **A** - **H** equal 20 μm while scale bar in **I** and **J** equal 10 μm.

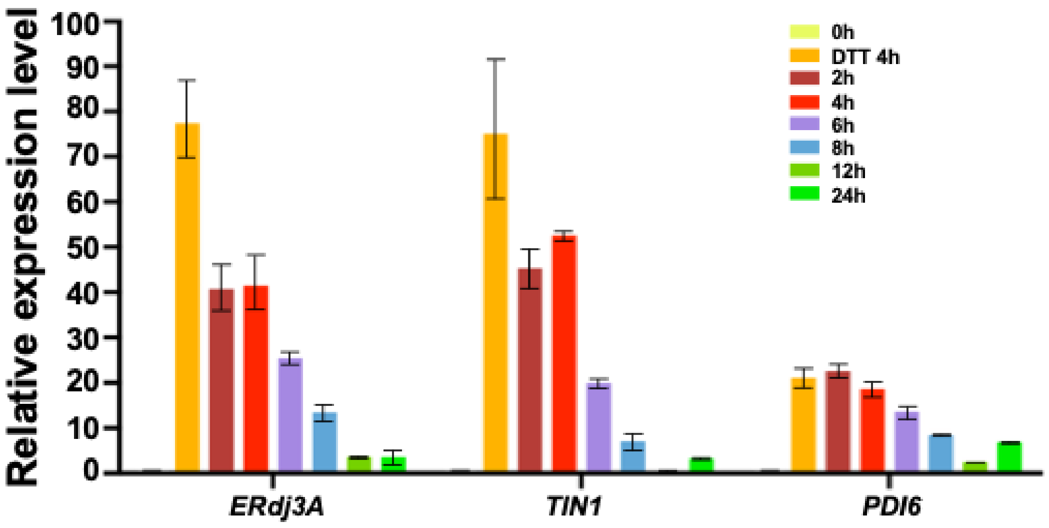

Supplementary Figure S12. Gradual reduction of the transcript abundance of three UPR genes following DTT removal

Reverse transcription-quantitative real-time PCR (RT-qPCR) analysis of the transcript abundance of three indicated UPR genes, *ERdj3A*, *TIN1*, and *PDI6*. Total RNAs were isolated from 10-day-old wild-type seedlings treated with or without 2 mM dithiothreitol (DTT) for 4 hours or DTT-treated seedlings incubated in the DTT-free ½ MS liquid medium for different durations. The isolated total RNAs were converted into the first-strand cDNA templates that were subsequently used to analyze the transcript abundance of the three indicated UPR genes via RT-qPCR using the RT-PCR primers listed in the **Supplementary Table S1**. The *ACTIN8* cDNA was used as the internal reference. Each bar indicates the average result of three biological replicates each with three technical repeats with error bars representing ±SD.

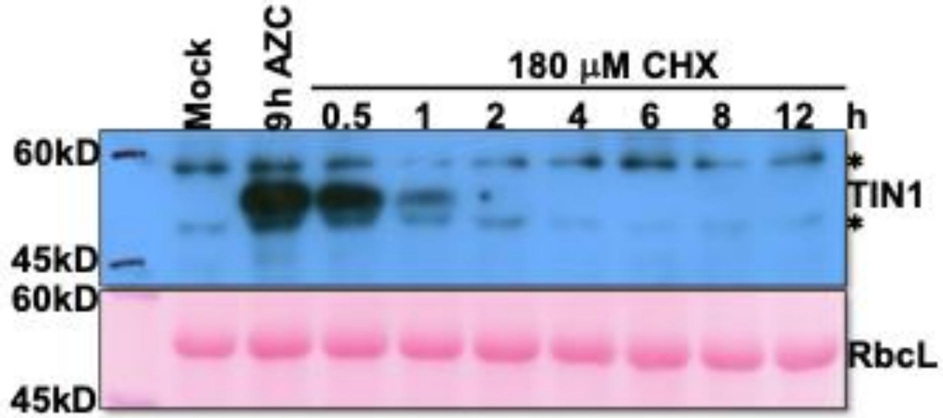

Supplementary Figure S13. The AZC-induced TIN1 protein is also rapidly degraded.
Immunoblot analysis of the TIN1 protein abundance in 2-week-old cycloheximide (CHX)-treated wild-type seedlings, which were pretreated with L-azetidine-2-carboxylic acid (AZC) or H_2O (Mock) for 4 hours followed by treatment with 180 μM CHX for different durations. Total proteins extracted from harvested Arabidopsis seedlings were separated by SDS-PAGE and analyzed by immunoblotting with anti-TIN1 antibody. The ponceau red-stained RbcL band serves as the loading control. Asterisks indicate non-specific cross-reacting bands. The positions of molecular mass standards are shown to the left of images.

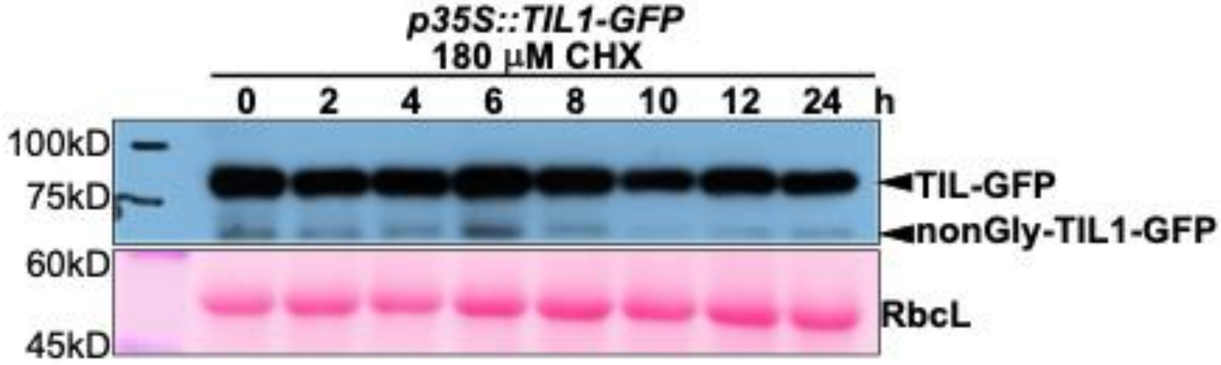

Supplementary Figure S14. TIL1 is a very stable protein.
Immunoblot analysis of the TIL1-GFP fusion protein in 10-day-old seedlings of the *p35S::TIL1-GFP* transgenic line treated with 180 mM cycloheximide (CHX) for indicated durations. The "nonGly" indicates the non-glycosylated form of TIN1, and the ponceau red-stained RbcL bands serve as the loading control. The positions of molecular mass standards are shown on the left of images.

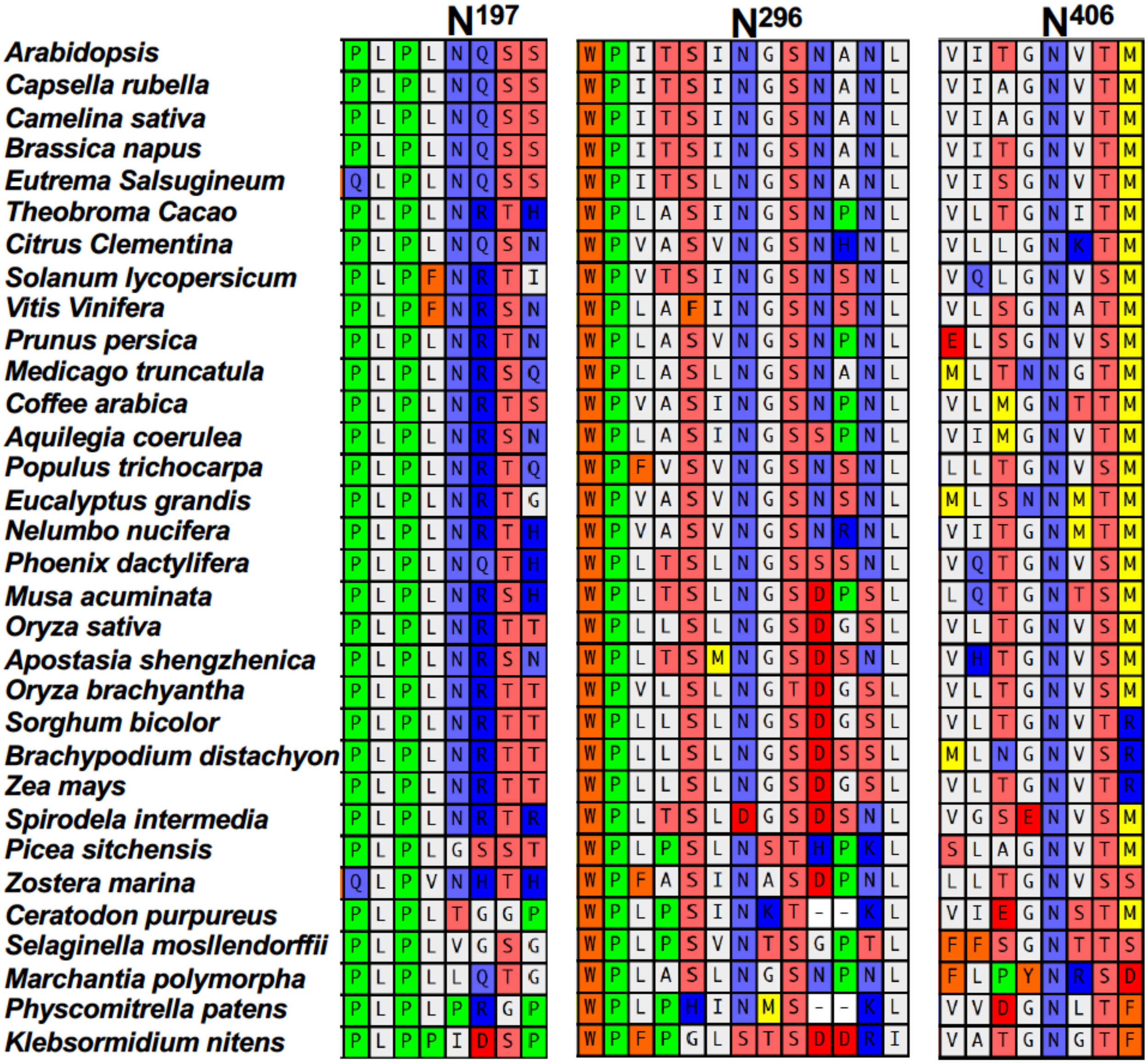

Supplementary Figure S15. Conservation of the three glycosylated Asn residues between TIN1 and its homologs in green organisms.

Shown here are three segment (surrounding the three glycosylated N residues) of the color-coded sequence alignment of TIN1 and its homologs. A multiple sequence alignment file, which was generated from the phylogeny analysis shown in **Supplementary Figure S5,** was visualized using MacVector (https://macvector.com/). Amino acids were colored using the chemical-type scheme: acidic, red; hydrophobic, gray; amido, blue; aromatic, orange; basic, deep blue; hydroxyl, salmon; proline, green; sulfur, yellow.

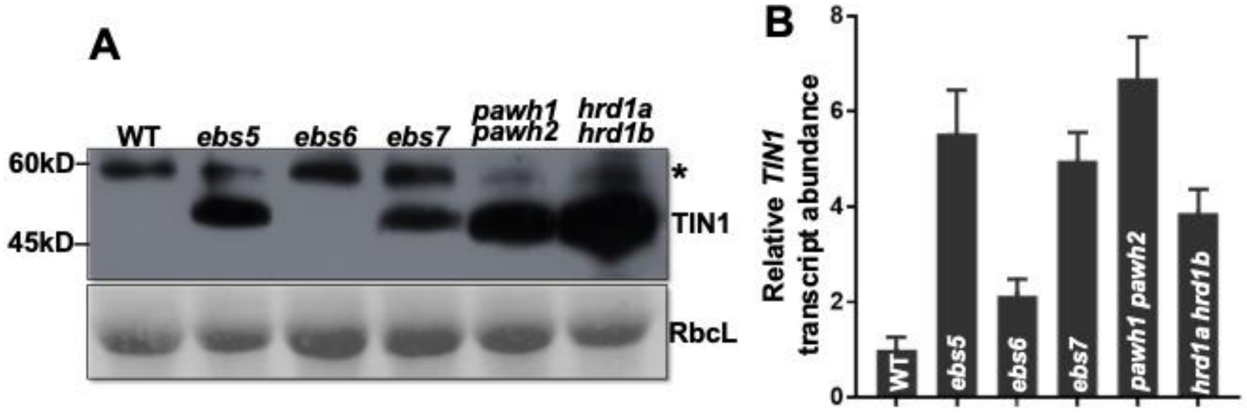

Supplementary Figure S16. The effect of the known ERAD mutations on the abundance of the *TIN1* transcript and TIN1 protein.

A. Immunoblot analysis of the TIN1 protein abundance in 10-day-old Arabidopsis seedlings of the wild-type and various ERAD mutants. It is interesting to note that TIN1 was not detectable in the *ebs6* mutant seedlings. The ponceau red-stained RbcL band serves as the loading control. Asterisks indicate non-specific cross-reacting bands. The positions of molecular mass standards are shown on the left of the immunoblot.

B. A RT-qPCR analysis of the *TIN1* transcript abundance in 10-day-old seedlings of the wild-type and various ERAD mutants. Each bar is the relative value of the abundance of a cDNA target in Arabidopsis seedlings of various ERAD mutants to that of the wild-type seedlings, which is the average of three biological replicates each with three technical repeats. The *ACTIN8* cDNA was used as the internal reference. Error bars denote ±SD.

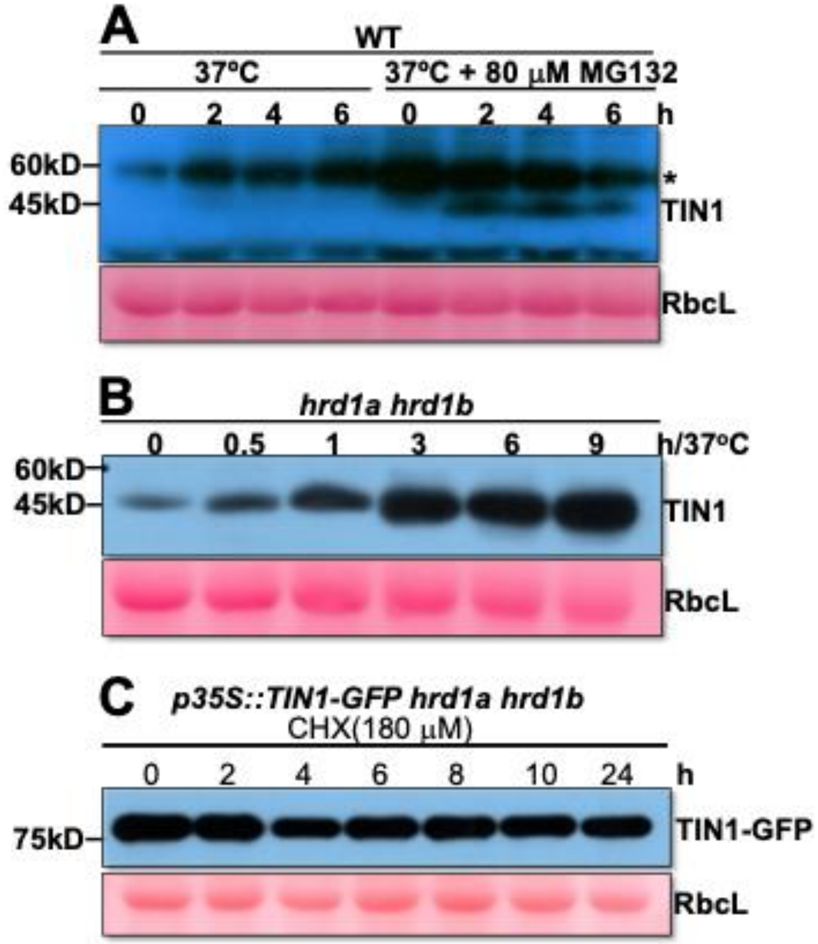

Supplementary Figure S17. The impact of heat stress, MG132, and *hrd1a hrd1b* double mutation on the TIN1 stability.

A. Immunoblot analysis of the TIN1 abundance in 10-day-old Arabidopsis seedlings treated with heat stress (for different durations) in the absence or presence of 80 μM MG132. The asterisk indicates the non-specific cross-reacting band.

B. Immunoblot analysis of the TIN1 abundance in 2-week-old seedlings of *hrd1a hrd1b* double mutant treated with the heat stress (for different durations).

C. Immunoblot analysis of the TIN1-GFP fusion protein in 10-day-old cycloheximide (CHX)-treated seedlings of a *p35S::TIN1-GFP hrd1 hrd1b* transgenic line for different durations. In **A**-**C**, the ponceau red-stained RbcL was used as the reference to control equal loading. The positions of molecular mass standards are shown to the left of the immunoblots.

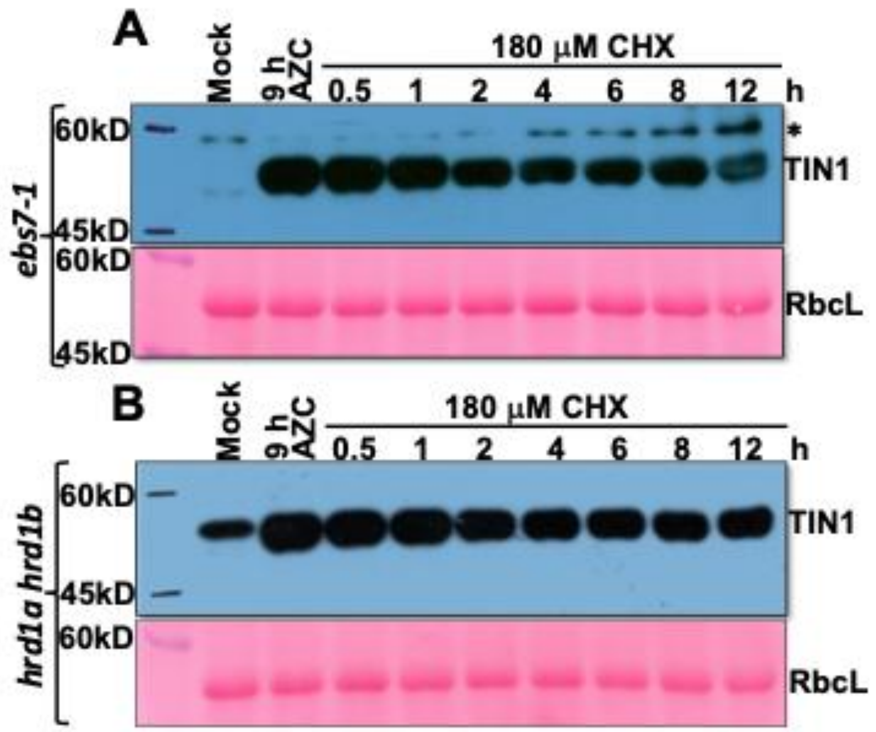

Supplementary Figure S18. The degradation of the AZC-induced TIN1 also involves the EBS7-PAWH1/2-Hrd1 machinery.

A, **B**. Immunoblot analysis of the TIN1 protein in 10-day-old seedlings of *ebs7-1* (A) and *hrd1a hrd1b* (**B**) mutants treated with 5 mM L-azetidine-2-carboxylic acid (AZC) or H_2O (mock) for 9 h followed by 180 μM cycloheximide (CHX) for different durations. The ponceau red-stained RbcL was used as the reference to control equal loading, while the star sign indicates the non-specific band. The positions of molecular mass standards are shown on the left of blot/gel images.

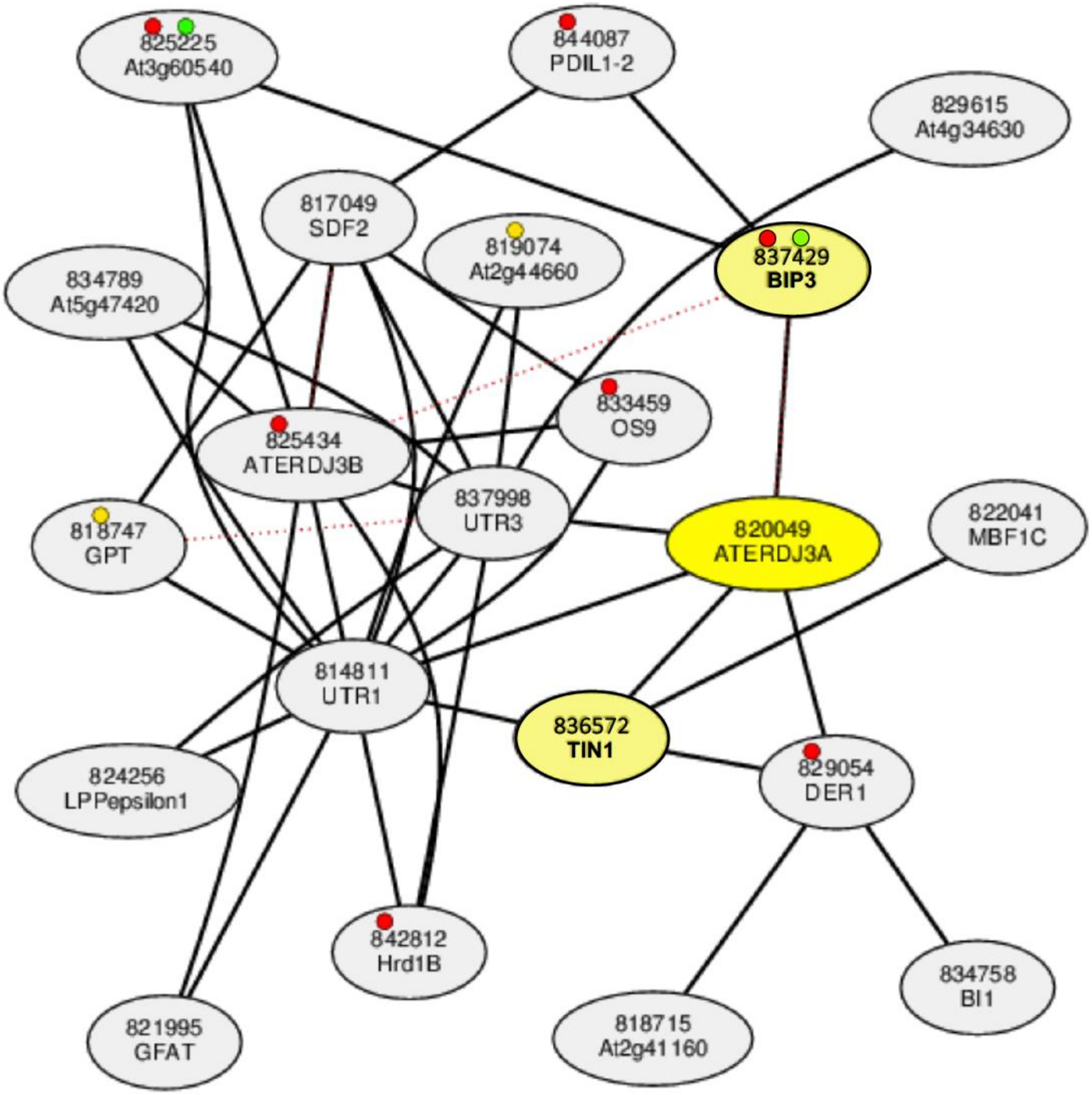

Supplementary Figure S19. *TIN1* is coexpressed with *BIP3* and *TMS1/ERdj3A*.
The image was obtained from the locus page of the search result using the gene ID (*At5G64510*) as the query at the ATTEDII site (http://atted.jp) (Obayashi et al., 2018). The three analyzed genes (*TIN1*, *At5G64510*; *BIP3*, *At1G09080*; *TMS1*)/*AtERdj3A*, *At3G08970*) were shaded with yellow color. Red, yellow, and green dots denote proteins predicted or experimentally proven to be involved in “protein processing in endoplasmic reticulum”, “N-glycan biosynthesis”, and “protein export”, respectively. Thick black lines indicate strongly-coexpressed genes while the dotted orange lines denote known protein-protein interactions.

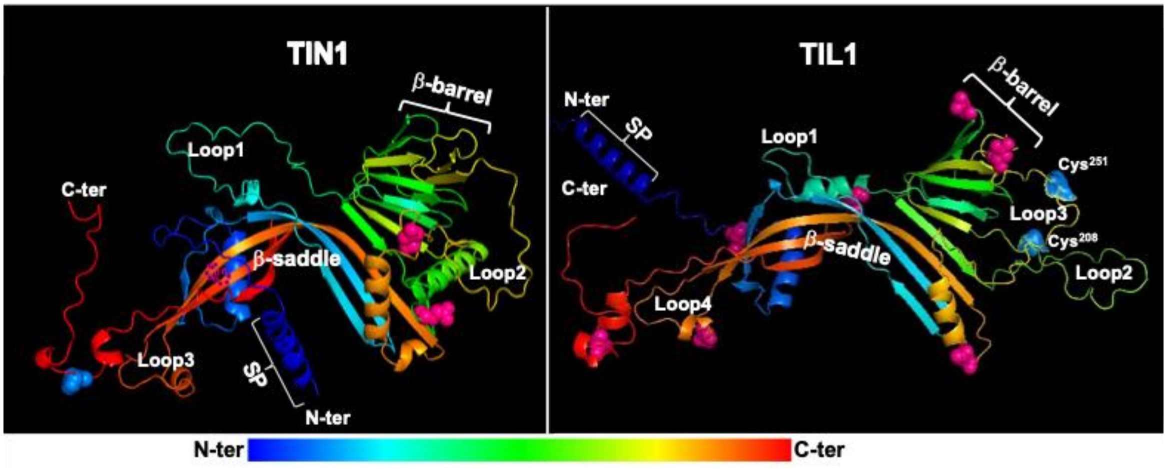

Supplementary Figure S20. AlphaFold-predicted structural models of TIN1 and TIL1
Shown here are rainbow-colored ribbon models of the AlphaFold-predicted three-dimensional structures of TIN1 (left) and TIL1 (right). The structural files, AF-A0A178UGX5-F1-v4 (TIN1) and AF-Q9FX03-F1-v4 (TIL1) were downloaded from https://alphafold.ebi.ac.uk/ (Jumper et al., 2021; Cheng et al., 2023), visualized and colored by PyMol (ver. 2.57) (https://www.pymol.org/). Both TIN1 and TIL1 have two major structural domains: a central b-saddle consisted of two symmetrically arranged b-sheets sharing a long-curved b-strand with their convex surfaces interacting with a single a-helix, and a lateral b-barrel with a hydrophobic cavity. The central b-saddle may be involved in protein-protein interaction while the lateral b-barrel may bind hydrophobic compounds. Each protein has a predicted signal peptide, indicated by a white bracket labelled as SP and three (TIN1) or four (TIL1) unstructured loops. The colored spherics mark the predicted N-glycosylation sites (three in TIN1 and seven in TIL1) while the light blue surfaces denote the two Cys residues in TIL1. N-ter indicates the N-terminal end while C-ter denotes the C-terminal tail of each protein. The rainbow bar indicates the color code used to mark the order of amino acid residues from the N-terminal head (blue) to the C-terminal tail (red).

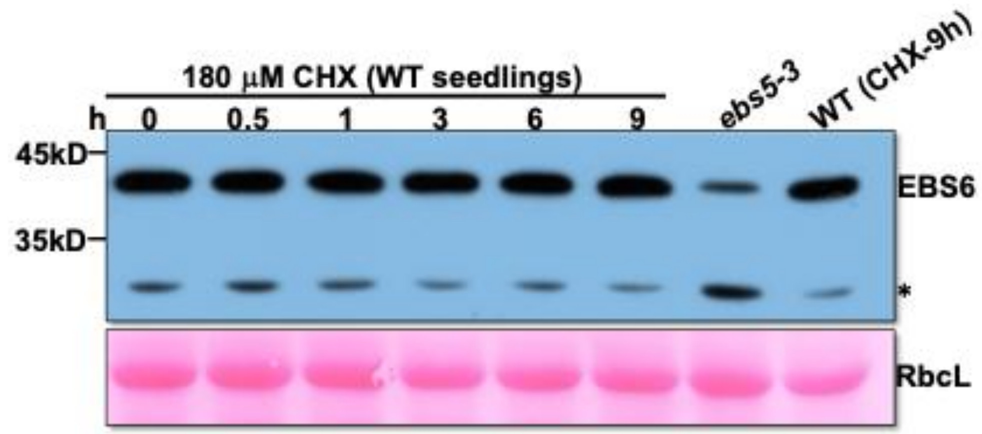

Fig. S21. The endogenous EBS6/AtOS9 is a very stable protein.

Immunoblot analysis of the protein abundance of EBS6 in 10-day-old seedlings treated with 180 μM cycloheximide (CHX) for varying durations. Total proteins from mock/CHX-treated seedlings and the *ebs5-3* mutant were extracted, separated by 10% SDS-PAGE, and analyzed by immunoblotting with an anti-EBS6 (Su et al., 2012). The ponceau red-stained RbcL serves as the loading control, while the asterisk denotes a non-specific band. The positions of molecular mass standards are shown to the left of the blot.

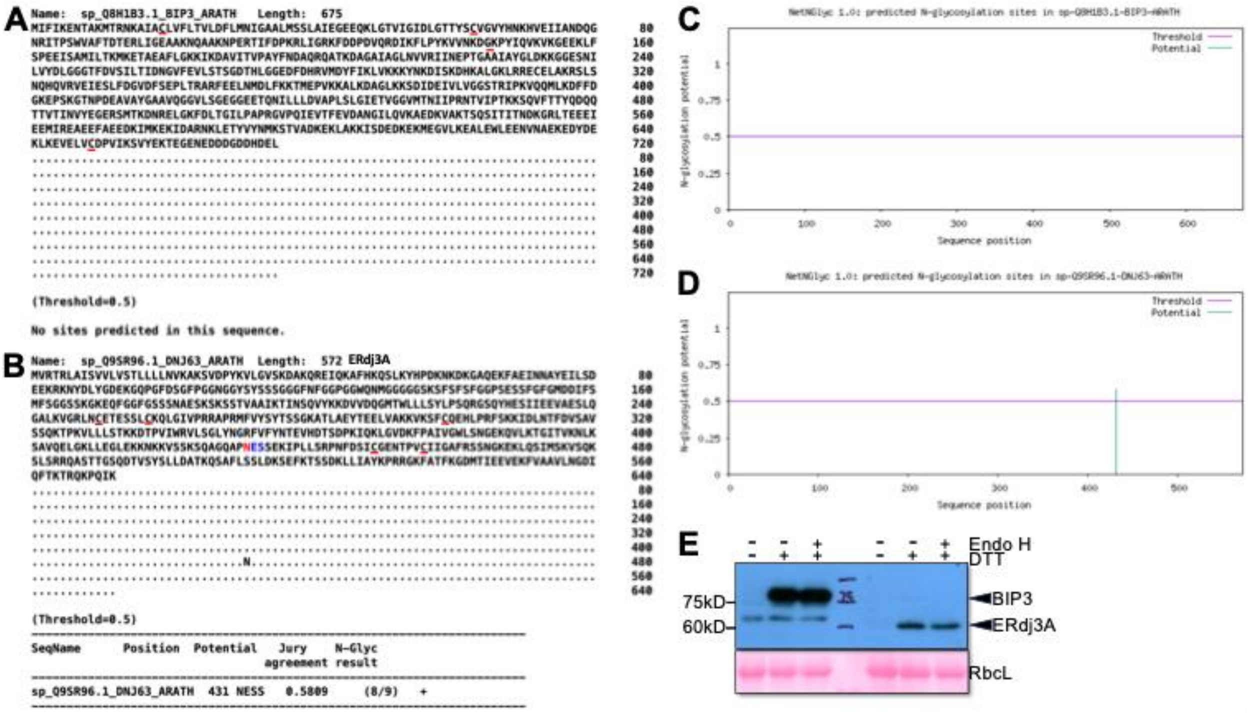

Supplementary Figure S22. Both BIP3 and ERdj3A are non-glycosylated ER proteins.

A-D. The screen shots of the N-glycosylation prediction results of BIP3 and ERdj3A (using the NetNGlyc-1.0 at https://services.healthtech.dtu.dk/services/NetNGlyc-1.0/ (Gupta and Brunak, 2002)**.** The prediction algorithm detects zero and one N-glycosylation site for BIP3 and ERdj3A, respectively. The red colored N indicates the predicted N-glycosylation site within the conserved NXS/T (NES) sequon while the red underlines mark four and five cysteine residues in BIP3 and ERdj3A, respectively.

E. Endo H analysis of the dithiothreitol (DTT)-induced BIP3 and ERdj3A. The first three lanes also contain a faint non-specific cross-reacting band while the ponceau red-stained RbcL bands serve as the loading control. The positions of molecular mass standards are shown on the left of the immunoblot.

References:

Cheng, J., Novati, G., Pan, J., Bycroft, C., Žemgulytė, A., Applebaum, T., Pritzel, A., Wong, L.H., Zielinski, M., Sargeant, T., Schneider, R.G., Senior, A.W., Jumper, J., Hassabis, D., Kohli, P., and Avsec, Ž. (2023). Accurate proteome-wide missense variant effect prediction with AlphaMissense. Science **381,** eadg7492.

Fucile, G., Di Biase, D., Nahal, H., La, G., Khodabandeh, S., Chen, Y., Easley, K., Christendat, D., Kelley, L., and Provart, N.J. (2011). ePlant and the 3D data display initiative: integrative systems biology on the world wide web. PLoS One **6,** e15237.

Gupta, R., and Brunak, S. (2002). Prediction of glycosylation across the human proteome and the correlation to protein function. Pac Symp Biocomput**,** 310-322.

Jumper, J., Evans, R., Pritzel, A., Green, T., Figurnov, M., Ronneberger, O., Tunyasuvunakool, K., Bates, R., Žídek, A., Potapenko, A., Bridgland, A., Meyer, C., Kohl, S.A.A., Ballard, A.J., Cowie, A., Romera-Paredes, B., Nikolov, S., Jain, R., Adler, J., Back, T., Petersen, S., Reiman, D., Clancy, E., Zielinski, M., Steinegger, M., Pacholska, M., Berghammer, T., Bodenstein, S., Silver, D., Vinyals, O., Senior, A.W., Kavukcuoglu, K., Kohli, P., and Hassabis, D. (2021). Highly accurate protein structure prediction with AlphaFold. Nature **596,** 583-589.

Lemoine, F., Correia, D., Lefort, V., Doppelt-Azeroual, O., Mareuil, F., Cohen-Boulakia, S., and Gascuel, O. (2019). NGPhylogeny.fr: new generation phylogenetic services for non-specialists. Nucleic Acids Res **47,** W260-W265.

Obayashi, T., Aoki, Y., Tadaka, S., Kagaya, Y., and Kinoshita, K. (2018). ATTED-II in 2018: A Plant Coexpression Database Based on Investigation of the Statistical Property of the Mutual Rank Index. Plant Cell Physiol **59,** 440.

Schwacke, R., and Flügge, U.-I. (2018). Identification and Characterization of Plant Membrane Proteins Using ARAMEMNON. In Plant Membrane Proteomics: Methods and Protocols, H.-P. Mock, A. Matros, and K. Witzel, eds (New York, NY: Springer New York), pp. 249-259.

Su, W., Liu, Y., Xia, Y., Hong, Z., and Li, J. (2012). The Arabidopsis homolog of the mammalian OS-9 protein plays a key role in the endoplasmic reticulum-associated degradation of misfolded receptor-like kinases. Mol Plant **5,** 929-940.

Teufel, F., Almagro Armenteros, J.J., Johansen, A.R., Gíslason, M.H., Pihl, S.I., Tsirigos, K.D., Winther, O., Brunak, S., von Heijne, G., and Nielsen, H. (2022). SignalP 6.0

predicts all five types of signal peptides using protein language models. Nature Biotechnology **40,** 1023-1025.

Yamaguchi-Shinozaki, K., and Shinozaki, K. (1994). A novel cis-acting element in an Arabidopsis gene is involved in responsiveness to drought, low-temperature, or high-salt stress. Plant Cell **6,** 251-264.

Supplementary Table S1. Oligonucleotide primers used in this study

Oligonucleotides/usages	Sequence
**Genotyping primers**	
tin1-1 (cs411789)-LP	ATGGGTCACAGAGTATTGGTTTATG
tin1-1 (cs411789)-RP	TGGTAAAGAAAGGCGCATATTATTAGC
tin1-1 (cs411789)-LB	CCCATTTGGACGTGAATGTAGACAC
til1-t (cs808001)-LP	ACATCGTATTCGGAAGTGTGC
til1-t (cs808001)-RP	GAAAGCGAGAGACGATCATTG
ebs5 (salk_006503)-LP	TTTACCATTTGGACGCAGATC
ebs5 (salk_006503)-RP	TGAACATGGACAAGGACTTCC
ebs6 (salk_029413)-LP	TCTATGATTTTGGCGTTTTGG
ebs6 (salk_029413)-RP	TTCCTTGTTTCTTGTTGCGTC
ebs7 dCAPs (PstI)-F	ATCATAGCTATACGTAACTG
ebs7 dCAPs (PstI)-R	CTTTCCACAACATGCCTTTGATCTG
hrd1a (salk_032914)-LP	CTTGAGCTTATCCGTGACCTG
hrd1a (salk_032914)-RP	TGCTACTGTGTTTGCAGATGG
hrd1b (salk_061776)-LP	AGTGGCATCATTTCTGCAAAC
hrd1b (salk_061776)-RP	GGAAGGGCTCAGGTGATTAAG
ire1a (salk_018112)-LP	TGCGTTCAGACACTAACATGC
ire1a (salk_018112)-RP	GAAGAAGAACCGTAAATCCGC
ire1b (sail_238_F07)-LP	CCTCTCGAACCCTTCAGGTAC
ire1b (sail_238_F07)-RP	GAAGGAAAACGGACATCCTTC
bzip28 (salk_132285)-LP	TTTATCATCATTTTGGTCGCC
bzip28 (salk_132285)-RP	TATCCCCTAACAGGATACGGC
bzip60 (sail_283_B03)-LP	CAAAGATTGGCTCGTCTGAAC
bzip60 (sail_283_B03)-RP	TGCTTGCGTATCTTGTGATATG
LBb1.3	ATTTTGCCGATTTCGGAAC
LB3	TAGCATCTGAATTTCATAACCAATCTCGATACAC
**qRT-PCR primers**	
qRT-TIN1-F	CTTTACCATATGATGTGGATGCTG
qRT-TIN1R	TGAAGTTGATGCAAGAGAAGTAGG
qRT-PDI6-F	TAGTGTACGAAGGAGACAGG
qRT-PCR-R	GAAGAAGAGAAACGCTCCTA
qRT-ERdj3A-F	ACCTCTTCTGTCAAGACCA;
qRT-ERdj3A-R	TCCTGAGAACCTGTGGTTG
qRT-BIP3-F	CACGGTTCCAGCGTATTTCAAT
qRT-BIP3-R	ATAAGCTATGGCAGCACCCGTT
qRT-Actin-F	GGTAACATTGTGCTCAGTGGTGG
qRT-Actin-R	AACGACCTTAATCTTCATGCTGC
qRT-RD29A-F	CCCACCAAAGAAGAAACTGGAG
qRT-RD29A-R	GCGAATCCTTACCGAGAACAG
qRT-RD29B-F;	GGCGGGCAAAGCGAG
qRT-RD29B-R	TGCCCGTAAGCAGTAACAGATC
qRT-TIL1-F	TCGTTACCGATGAACATTTCAC
qRT-TIL1-R	GATTGCCTCATCTGATAAGTACG
**Transgenic primers**	
pTIN1::GUS-F	GCTGCAGCAAGATGGTAAAATATTACC
pTIN1::GUS-R	CGGATCCCTTGGATTGACCAAAAAAAACCAGGCC
cTIN1-GFP-F	ATTTGGAGAGGACAGGGTACCATGGGTCACAGAGTATTGGTTTATGT
cTIN1-GFP-R	AGTGTCGACTCTAGAGGATCCCAAGGTAAAAGGGCTTGGAGG
gTIN1(g)-GFP-F	TATGACCATGATTACGAATTCCAAATGCTTTTAAGGTAAGATCATCAA
gTIN1(g)-GFP-R	AGTGTCGACTCTAGAGGATCCCAAGGTAAAAGGGCTTGGAGG
gTIN1(g)-N197Q-F	TTTGCCACTTCCATTGCAACAGAGTTCCAATGAGTTTGCTTCT
gTIN1(g)-N197Q-R	AGAAGCAAACTCATTGGAACTCTGTTGCAATGGAAGTGGCAAA
gTIN1(g)-N296Q-F	TGTGGCCAATCACTTCAATCCAAGGCTCAAATGCTAACTTACT
gTIN1(g)-N296Q-R	AGTAAGTTAGCATTTGAGCCTTGGATTGAAGTGATTGGCCACA
gTIN1(g)-N406Q-F	CGCAAAATGTGATTACTGGACAAGTAACCATGTCGAAACTTCC
gTIN1(g)-N406Q-R	GGAAGTTTCGACATGGTTACTTGTCCAGTAATCACATTTTGCG
c-TIL1-GFP-F	ATTTGGAGAGGACAGGGTACCATGAAGATCTGTCGCTCTCCTTCC
c-TIL1-GFP-R	AGTGTCGACTCTAGAGGATCCCCATTTGACATCGAGTGTTAAAGC
**TIN1 antigen primers**	
TIN1ab-F	GGAATTCTCGTCTCCTGATGCTAATC
TIN1ab-R	GCGTCGACCAAGGTAAAAGGGCTTGGAG
**BiFC assay primers**	
TIN1-NE-F	CACTAGTATGGGTCACAGAGTATTG
TIN1-NE-R	GCGTCGACCAAGGTAAAAGGGCTTG
MNS4-CE-F	TGGCGCGCCACTAGTGGATCCATGGACTCAAATTTCAAGTGGCTT
